# Compact Occlusion-Robust Facial Expression Recognition via Clean-Anchored Hard Occlusion Fine-Tuning

**DOI:** 10.3390/s26175500

**Published:** 2026-08-30

**Authors:** Xuefeng Zhao, Yixuan Dong, Zhaoman Zhong, Tingchen Jiang

**Affiliations:** 1 School of Computer Engineering, Jiangsu Ocean University, Lianyungang 222005, China; 2024220908@jou.edu.cn (Y.D.); zmzhong@jou.edu.cn (Z.Z.); 2 School of Smart Ocean, Jiangsu Ocean University, Lianyungang 222005, China; 3 School of Marine Technology and Geomatics, Jiangsu Ocean University, Lianyungang 222005, China; jiangtc@jou.edu.cn

**Keywords:** visual affective sensing, facial expression recognition, occluded facial expression recognition, structured occlusion augmentation, teacher–student learning, compact inference

## Abstract

Facial occlusion removes expression-relevant evidence and remains a major source of error in camera-based affective sensing. Existing approaches to occlusion-robust facial expression recognition often rely on specialized attention, reconstruction, semantic, or geometric pipelines, whereas aggressive training using synthetic occlusions may impair discrimination on clean images or overfit to synthetic corruption patterns. We address this tension between cleanness and robustness through clean-anchored hard occlusion fine-tuning (CA-HOFT). HardMix samples structured and random occlusion modes according to a facial region-weighted distribution. An explicit classification branch for the non-HardMix source view preserves ground-truth supervision, while a fixed reference teacher initialized from the preceding mixed-occlusion stage supplies a stationary distribution for both paired student views. These signals jointly train a single classifier while retaining a single-backbone inference pathway. Across five independent training runs, the ResNet-18 student achieves 90.08% accuracy on the Real-World Affective Faces Database (RAF-DB) and 87.38% accuracy with 83.34% macro-F1 on Occlusion-RAF-DB, with corresponding sample standard deviations of 0.21, 0.12, and 0.32 percentage points. The retained model has 11.18 million parameters and requires 1.814 giga multiply–accumulate operations (GMACs). Controlled comparisons and ablations indicate that it has the most favorable observed cleanness–robustness trade-off among the tested epoch-matched alternatives; however, fixed-checkpoint comparisons on Occlusion-RAF-DB are not significant after Holm correction. AffectNet-8 and evaluations using natural occlusion provide supporting evidence, while broader cross-domain validation remains future work.

## 1. Introduction

Vision-based facial expression recognition (FER) supports affective sensing in human–computer interaction, healthcare, driver assistance, education, and robotics. In this study, visual affective sensing refers specifically to static categorical FER rather than the direct inference of internal affective states. Although modern FER models perform well on in-the-wild datasets such as the Real-World Affective Faces Database (RAF-DB) and AffectNet, expression-critical evidence can be obscured by masks, hands, hair, glasses, or head-mounted displays. A practical model must improve robustness to such occlusions without sacrificing discrimination on clean images or requiring a costly occlusion-specific inference pipeline. We assess this balance using absolute performance on clean and naturally occluded images rather than the clean-to-occlusion drop alone, as a smaller drop may simply reflect a weaker clean model.

Existing approaches reweight unreliable regions [1,2], reconstruct missing content [3], or incorporate semantic and geometric priors [4,5,6]. Although these mechanisms can improve robustness, they may require additional detectors, reconstruction modules, or prior extractors. Training-time erasing offers a simpler alternative, but generic masks disregard facial structure, and strong synthetic corruption can displace the classifier from its clean-image decision function. Conventional single-view distillation also fails to explicitly couple an occluded student prediction to the reference distribution obtained from the corresponding source image.

To address this, we investigate whether hard occlusion learning can remain anchored to clean-image semantics. Clean-anchored hard occlusion fine-tuning (CA-HOFT) combines an expression region-aware coarse HardMix policy with an explicit clean-label classification branch and a fixed reference teacher. The teacher is initialized from the preceding mixed-occlusion fine-tuning (Mixed FT) stage and evaluated only on the non-HardMix source input. In this paper, clean anchoring refers to the joint preservation of direct label supervision and the teacher’s fixed reference distribution on the non-HardMix view. A cross-view constraint transfers this reference to the HardMix student view. A single ResNet-18 student learns from all of these constraints, thereby preserving a compact single-backbone deployment pathway. Here, “compact” means that inference retains the standard 11.18M-parameter graph without an occlusion-specific module; it does not mean that we introduce a new lightweight backbone.

The main contributions of this paper are summarized as follows:

- We introduce a staged clean-anchored hard occlusion training formulation that couples expression region-aware coarse HardMix with explicit clean-label supervision and fixed-reference consistency on paired non-HardMix and HardMix views.
- We separately examine the roles of the clean-label term and the two teacher constraints through controlled baselines in the final stage and schedule-matched component ablations.
- We characterize robustness across occluder appearances and datasets using a testing-only benchmark of held-out alpha-composited objects, synthetic severity tests, and natural occlusion benchmarks. We further quantify statistical uncertainty and deployment cost through paired analyses and network-only inference profiling.

The multi-run controlled comparisons and schedule-matched RAF-DB ablations provide the primary evidence; analyses of severity, natural occlusion, AffectNet-8, and the Facial Expression Dataset with Real-World Occlusion (FED-RO) along with paired and literature analyses provide supporting context.

## 2. Related Work

### 2.1. In-the-Wild and Deployment-Oriented FER

In-the-wild FER is challenged by pose, illumination, identity, acquisition conditions, annotation uncertainty, and class ambiguity. The Self-Cure Network estimates sample reliability to suppress uncertain examples [7]. Learning with inconsistently annotated datasets explicitly models supervision disagreement across datasets [8], while multi-task assisted correction uses auxiliary tasks to refine uncertain labels [9]. Deep Attentive Center Loss improves class separation through attention-guided center learning [10], whereas semantic neighborhood-aware learning preserves relationships among expression categories [11]. POSTER uses pyramid cross-fusion for transformer-based FER [12]; POSTER++ simplifies the transformer design while strengthening recognition [13]; CLIPER introduces unified vision–language supervision [14]; and a dual-direction attention long short-term memory (LSTM) network combines temporal mixed features with CLIP [15]. SynFER expands FER training data with synthetic samples [16]. For resource-constrained deployment, EfficientFace uses lightweight label distribution training [17], mobile affective computing targets real-time on-device inference [18], Light-FER targets edge devices [19], and spatial bias designs reduce the cost of expression recognition [20].

These representative directions improve label handling, feature selectivity, semantic supervision, data scale, or deployment cost, but generally assume that expression-relevant facial evidence remains available. They do not directly address the failure mode considered here, namely, that physical occlusion can remove such evidence at inference. We do not propose another general FER backbone or lightweight architecture; instead, we examine whether training-stage constraints can preserve a clean decision function while the model learns from a paired input that deliberately removes facial evidence.

### 2.2. Occlusion-Robust Facial Expression Recognition

Methods for occlusion-robust FER differ in how they handle unreliable or missing evidence. GACNN uses an attention mechanism to reduce the influence of occluded facial areas [21]. The Region Attention Network (RAN) aggregates region-level predictions to emphasize visible cues [1], whereas the Occlusion-Adaptive Deep Network (OADN) adapts its representation to the detected occlusion pattern [2]. This family demonstrates the value of spatially selective processing, but introduces mechanisms specialized for identifying or reweighting facial regions.

A second family models the missing information or incorporates explicit facial priors. Latent-OFER reconstructs a latent representation of the occluded face [3]. ORSANet introduces semantic-aware occlusion modeling [4]; Mukhiddinov et al. combined facial landmarks with deep learning for emotion recognition of masked faces [5]; and So and Han used landmark-driven keypoint features [6]. Kim and Lee addressed occlusion and intra-class similarity through relevant subsampling [22], while Wang et al. used self-supervised learning to obtain occlusion-tolerant features [23]. Studies involving head-mounted displays further show that systematic loss of facial regions remains a practical sensing problem: EmojiHeroVR examines partial occlusion from head-mounted displays [24], while EmoHeVRDB evaluates static FER for virtual reality users [25]. While these approaches can improve robustness, their reliance on reconstruction, auxiliary priors, specialized modules, or alternative representation objectives creates different training and deployment trade-offs. The present study addresses the narrower question of whether training stage constraints can improve tolerance to missing evidence while keeping the deployment an otherwise standard ResNet-18.

### 2.3. Occlusion Augmentation and Reference-Preserving Learning

Training-time corruption exposes classifiers to incomplete evidence without adding an occlusion-specific inference module. Random erasing removes randomly sampled regions [26], cutout masks a contiguous image block [27], and CutMix replaces a region with content from another training image [28]. Erasing-based FER consistency further stabilizes predictions under perturbation [29]. These methods establish the value of corrupted views, but their generic regions are not designed around expression-relevant facial evidence; stronger corruption may improve tolerance to missing evidence while disrupting the clean-view decision function.

Knowledge distillation and consistency regularization constrain predictive drift through several related mechanisms. Classical distillation transfers softened teacher probabilities to a student [30]; a later survey organized the main distillation families [31]. Learning Without Forgetting retains responses from a previous model during adaptation [32], while a Born-Again Network uses a predecessor model as a self-distillation teacher [33] and Mean Teacher uses an exponential-moving-average target [34]. Unsupervised data augmentation enforces prediction consistency across augmented views [35], Noisy Student combines teacher-generated targets with stronger student noise [36], and FixMatch couples confidence-thresholded pseudolabels with weak/strong augmentation [37]. These formulations motivate reference preservation, but do not directly couple a deliberately hard-occluded student view to a fixed non-HardMix reference from the same labeled source face.

This missing connection motivates CA-HOFT. The preceding mixed FT checkpoint provides a fixed reference after moderate exposure to occlusion. For each labeled source face, the teacher distribution on the non-HardMix view constrains both the student’s non-HardMix prediction and its paired HardMix prediction, while ground-truth classification remains active for both student views. The resulting formulation combines targeted evidence removal with same-source reference preservation instead of treating augmentation and distillation as independent operations. Pair construction, occlusion generation, and teacher supervision are confined to training; deployment retains only the ResNet-18 student.

## 3. Materials and Methods

### 3.1. Problem Formulation and Method Overview

**Figure 1:**
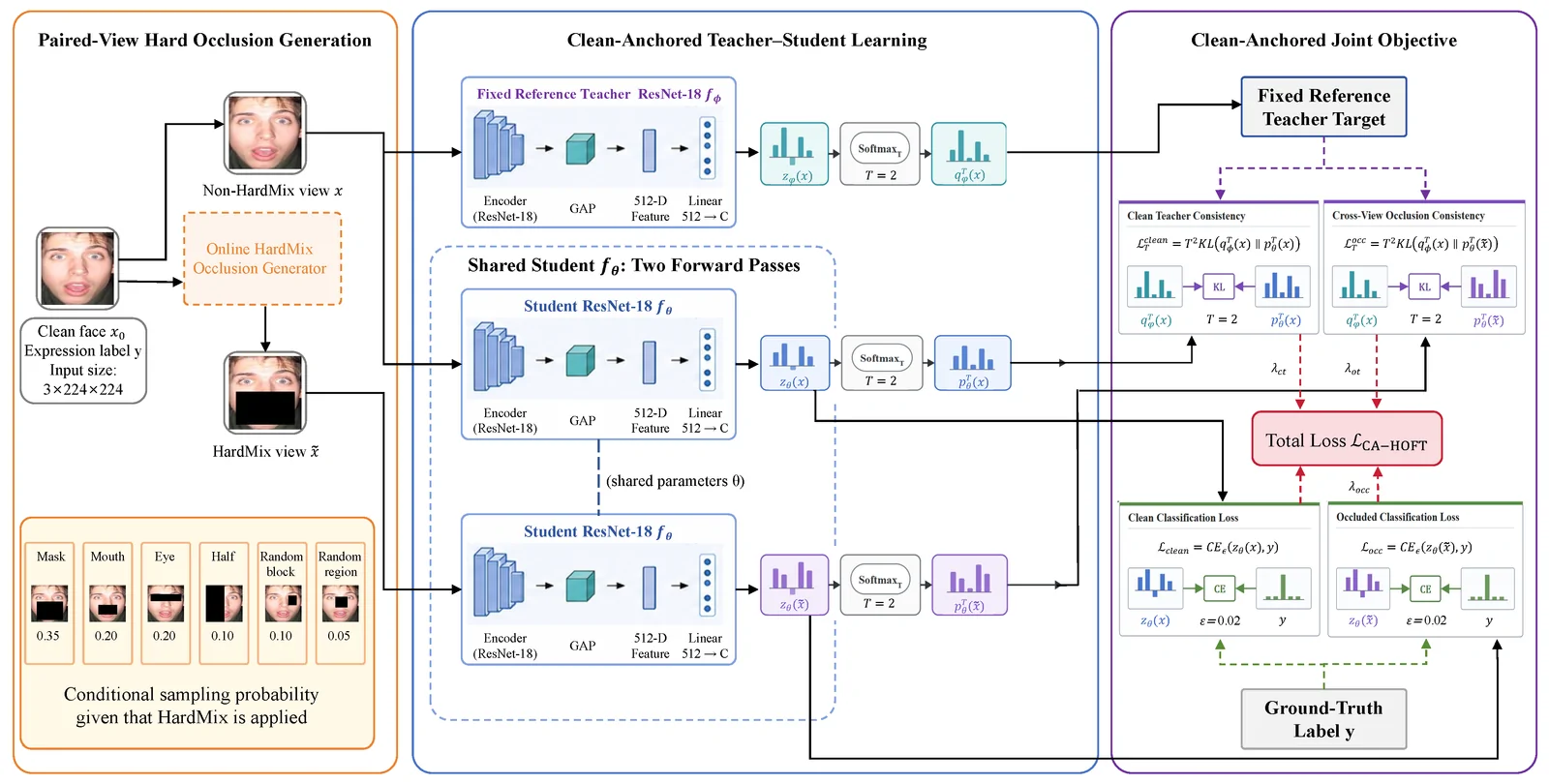
Overview of clean-anchored hard occlusion fine-tuning (CA-HOFT). An illustrative labeled training set source face $x_{0}$ yields a non-HardMix view *x* and paired HardMix view $\tilde{x}$ for one shared student. The fixed teacher from the preceding mixed-occlusion fine-tuning (mixed FT) stage evaluates only *x* and supplies the reference to both student predictions; ground-truth classification remains active on both branches. In the schematic, “clean face” denotes the labeled source face and “mask” denotes the mask-like HardMix mode. The thumbnails are schematic; implemented masks follow Section 3.2. Only the student remains at inference.

Our objective is to improve robustness to missing facial evidence while limiting degradation of the decision function learned before hard-occlusion fine-tuning. Figure 1 summarizes the resulting training pipeline. Let $x_{0}\in X$ denote an aligned source face with expression label y∈{1,…,C}. After applying a shared horizontal flip, we construct two student views from the same source image. The upper branch applies the non-HardMix augmentation pipeline

(1) $$x=A_{c}\left(x_{0}\right),$$

whereas the lower branch first applies a targeted occlusion $O∼Q_{O}$ and then its branch-specific augmentation

(2) $$\tilde{x}=A_{o}\left(O\left(x_{0}\right)\right).$$

Thus, *x* and $\tilde{x}$ originate from the same image and share the same class label, but independently sampled branch transformations need not preserve pixelwise registration. We use the term “non-HardMix” deliberately: the targeted HardMix operator is not applied to *x*, although its generic augmentation pipeline may still include low-probability random erasing. Consequently, the cross-view constraint regularizes the combined change induced by HardMix and the independently sampled branch augmentations; it should not be interpreted as isolating the effect of HardMix alone.

At the center of Figure 1, a single ResNet-18 student $f_{\theta }$ processes both views using shared parameters. Its logits and temperature-scaled posterior are denoted by $z_{\theta }\left(\cdot \right)=f_{\theta }\left(\cdot \right)$ and $p_{\theta }^{T}\left(\cdot \right)=\mathrm{softmax}\left(z_{\theta }\left(\cdot \right)/T\right)$, respectively. A reference teacher $f_{\phi }$ is initialized from the seed-matched mixed FT checkpoint, remains in evaluation mode with parameters and normalization statistics fixed, and receives only *x*. This checkpoint is obtained before HardMix fine-tuning but already reflects moderate exposure to occlusion; its use is a stage-wise design choice.

The right side of Figure 1 shows the four supervision paths. Ground truth classification is applied separately to the student’s predictions for *x* and $\tilde{x}$. In parallel, the teacher distribution computed from *x* is detached and used twice: first to constrain the student’s prediction for the non-HardMix view, then to regularize the paired HardMix prediction toward the same source-specific reference. This design avoids requiring the teacher to interpret the occluded input directly, while the cross-view term spans all differences between the independently augmented branches rather than an occlusion-only intervention. Section 3.3 formalizes the two classification losses and two teacher consistency losses.

HardMix increases exposure to missing facial evidence, while label supervision and the fixed source-view reference constrain the shared student. The teacher, paired-view construction, HardMix generator, and losses are excluded from deployment, which uses only the updated ResNet-18 student.

### 3.2. Staged Initialization and HardMix Generation

The first stage, Strong Clean, establishes the initial discriminative model without a paired facial occlusion branch. Its generic augmentation pipeline may include low-probability random erasing. Accordingly, “Clean” indicates the absence of the dedicated paired occlusion objective, not the absence of all pixel-level occlusion in the augmented training inputs. With label-smoothing coefficient ϵ, the cross-entropy (CE) objective is

(3) $$L_{\mathrm{clean}}={\mathrm{CE}}_{\epsilon }\;\left(z_{\theta }\left(x\right),y\right),$$

where ${\mathrm{CE}}_{\epsilon }$ uses $s_{\epsilon }\left(y\right)=\left(1-\epsilon \right)e_{y}+\left(\epsilon /C\right)1$. The final Strong Clean checkpoint initializes mixed FT.

In the second stage, mixed FT introduces a paired occluded view before applying the harder HardMix policy. When the occluder is activated, this broader mixed policy samples uniformly among mouth, eye, brow, nose, mask-like, random-region, half-face, and random-block modes. The student is optimized using

(4) $$L_{\mathrm{mixed}}=L_{\mathrm{clean}}+\lambda_{\mathrm{occ}}L_{\mathrm{occ}},\;L_{\mathrm{occ}}={\mathrm{CE}}_{\epsilon }\;\left(z_{\theta }\left(\tilde{x}\right),y\right).$$

Mixed FT broadens the perturbation distribution before HardMix concentrates training on a harder policy weighted by facial region. Its final checkpoint then initializes the CA-HOFT student and fixed reference teacher.

HardMix is a non-learned stochastic online transformation with fixed region definitions and predefined sampling probabilities. Its emphasis on facial regions is motivated by evidence that spatially selective processing benefits occlusion-robust FER [1,2,21]. The term HardMix denotes a probabilistic mixture of hard-occlusion modes; it does not mix pixels or labels across training images. HardMix defines normalized boxes for the brow (0.12,0.16,0.88,0.34), eye (0.12,0.28,0.88,0.52), nose (0.28,0.40,0.72,0.72), and mouth (0.18,0.62,0.82,0.90), where each tuple gives $\left(x_{1},y_{1},x_{2},y_{2}\right)$ relative to the image width and height. A black rectangle covers the selected region. This coordinate-based prior requires no landmarks, segmentation, or learned localization.

The hard policy contains six modes:

(5) $$K_{\mathrm{hard}}=\left\{\text{mask-like},\mathrm{mouth},\mathrm{eye},\text{half-face},\mathrm{random}\;\mathrm{block},\mathrm{random}\;\mathrm{region}\right\}.$$

Conditioned on activating the occluder, their sampling probabilities (listed in the same order as in Equation (5)) are

(6) $$q_{\mathrm{hard}}=\left(0.35,0.20,0.20,0.10,0.10,0.05\right).$$

Mask-like occlusion uses (0.12,0.55,0.88,0.98), while half-face occlusion covers the left or right half with equal probability. For random-block occlusion, a side-length fraction *s* is sampled uniformly from [0.22,0.40]. On a square 224×224 input, the nominal integer width and height are obtained by truncating 224 s. The top-left integer coordinate is then sampled uniformly subject to $x_{1}+w\leq 224$ and $y_{1}+h\leq 224$. Consequently, the black block is generated entirely within the image rather than placed outside the image and then cropped. Random-region occlusion does not introduce another geometry; instead, it uniformly re-selects one of the four fixed brow, eye, nose, or mouth boxes defined above. This mechanism provides low-probability coverage of the brow and nose even though neither appears as a separate mode in Equation (5), and gives the eye and mouth a small additional probability through the random-region path. Because the probability of random-region occlusion is 0.05, it contributes 0.0125 to each of the four regions. The resulting effective conditional probabilities from the dedicated and random-region paths are 0.2125 for the eye, 0.2125 for the mouth, 0.0125 for the brow, and 0.0125 for the nose. It should be noted that the six sampling modes do not make for six geometrically disjoint occlusion types. Whenever the occluder is activated, a fresh online occlusion type is sampled; the stochastic choices within the half-face, random-block, and random-region modes are resampled as well. The training schedule controls the activation probability $p_{\mathrm{occ}}$, whereas Equation (6) defines the conditional distribution over occlusion types.

### 3.3. Clean-Anchored Teacher–Student Objective

For each paired input, the student produces $z_{\theta }\left(x\right)$ and $z_{\theta }\left(\tilde{x}\right)$, while the fixed teacher produces the detached reference distribution for *x*:

(7) $$q_{\phi }^{T}\left(x\right)=\mathrm{SG}\;\left[\mathrm{softmax}\;\left(z_{\phi }\left(x\right)/T\right)\right]$$

where SG denotes stop-gradient. The detached output provides stationary soft interclass relationships throughout CA-HOFT.

The clean-view consistency term uses the Kullback–Leibler (KL) divergence to preserve the teacher’s soft class relationships on the non-HardMix student input:

(8) $$L_{T}^{\mathrm{clean}}=T^{2}\mathrm{KL}\;\left(q_{\phi }^{T}\left(x\right)\;∥\;p_{\theta }^{T}\left(x\right)\right)$$

while the cross-view term transfers the same reference distribution to the occluded student prediction,

(9) $$L_{T}^{\mathrm{occ}}=T^{2}\mathrm{KL}\;\left(q_{\phi }^{T}\left(x\right)\;∥\;p_{\theta }^{T}\left(\tilde{x}\right)\right).$$

We use the teacher-to-student direction, $q_{\phi }^{T}\left(x\right)$ is detached, and $T^{2}$ preserves the distillation gradient scale [30].

Combining the four terms gives

(10) $$L_{\text{CA-HOFT}}=L_{\mathrm{clean}}+\lambda_{\mathrm{occ}}L_{\mathrm{occ}}+\lambda_{\mathrm{ct}}L_{T}^{\mathrm{clean}}+\lambda_{\mathrm{ot}}L_{T}^{\mathrm{occ}}.$$

**Figure 2:**
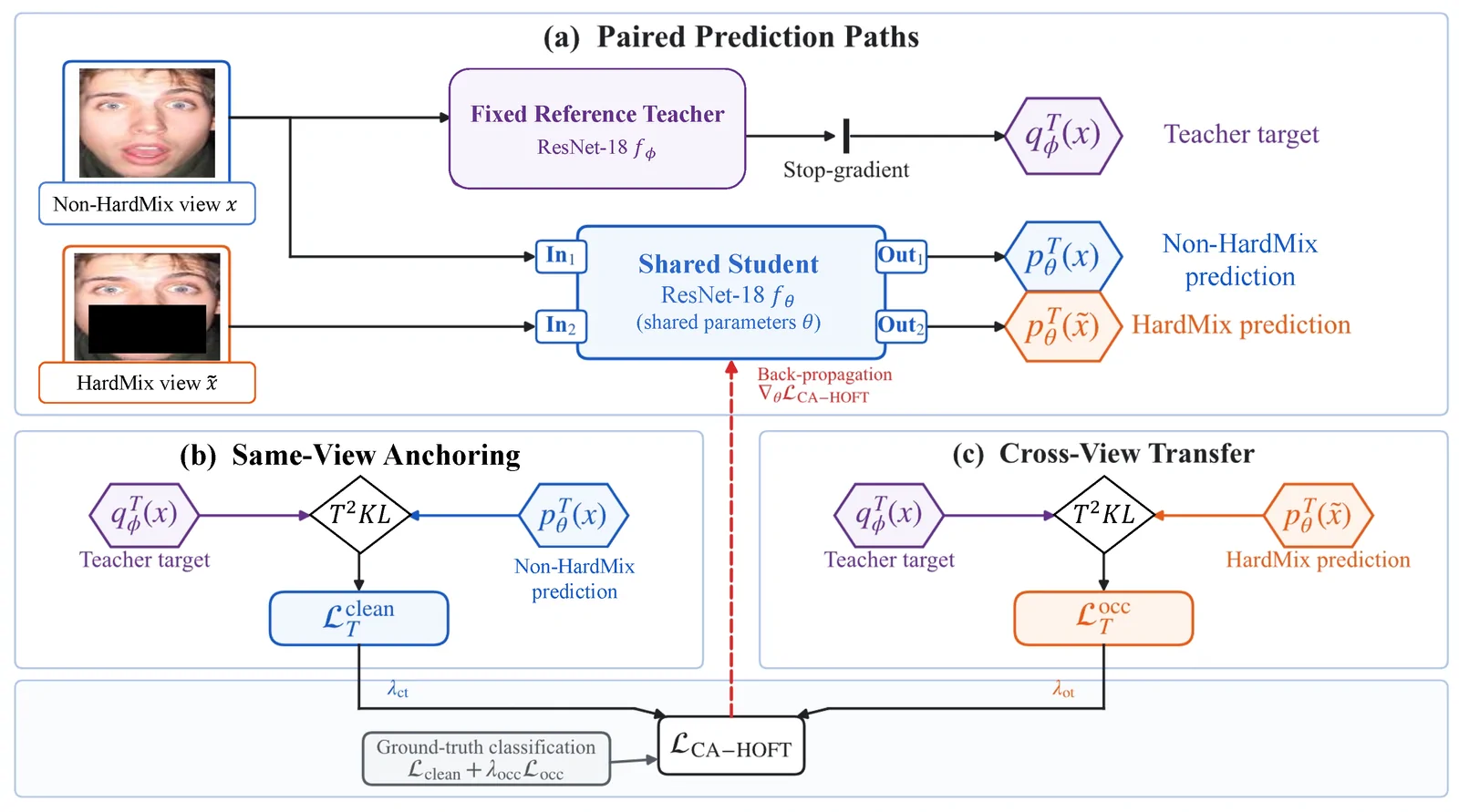
Objective for clean-anchored hard occlusion fine-tuning (CA-HOFT). The fixed teacher supplies $q_{\phi }^{T}\left(x\right)$ to both student predictions through KL divergence. Panel (**b**) preserves the reference on *x*, whereas panel (**c**) transfers it to $\tilde{x}$. The two classification losses and two consistency losses update only the shared student. Thumbnails are schematic, and the independently augmented branches need not remain pixel-wise registered.

The four terms enforce clean-label classification, HardMix classification, clean-view reference preservation, and cross-view reference transfer, respectively. We call $L_{\mathrm{clean}}$ the “clean-label anchor” and the detached teacher distribution the “clean-view reference”, which together define clean anchoring in CA-HOFT. These two clean-anchoring mechanisms are complementary rather than redundant. Cross-entropy preserves the hard class target, whereas distillation retains the soft interclass structure of the preceding mixed FT model. Their non-negative coefficients are loss weights, and both views update the same decision function. Figure 2 summarizes the resulting information and gradient flows.

#### Recovery Fine-Tuning

Recovery fine-tuning initializes the student from the preceding CA-HOFT checkpoint and retains the fixed mixed FT reference teacher used during CA-HOFT. It uses the objective in Equation (10) with the milder schedule reported in Table 1. Recovery FT serves as a post hoc diagnostic fine-tuning experiment, and is not part of the default CA-HOFT training sequence or the primary model.

For completeness, Table 1 summarizes the stage-specific optimization schedule used in the main RAF-DB experiments. The table is placed here because these schedules define the staged training procedure; dataset splits, hardware, random seeds, and evaluation protocols are reported separately in Section 4.1.

### 3.4. Student-Only Inference

**Figure 3:**
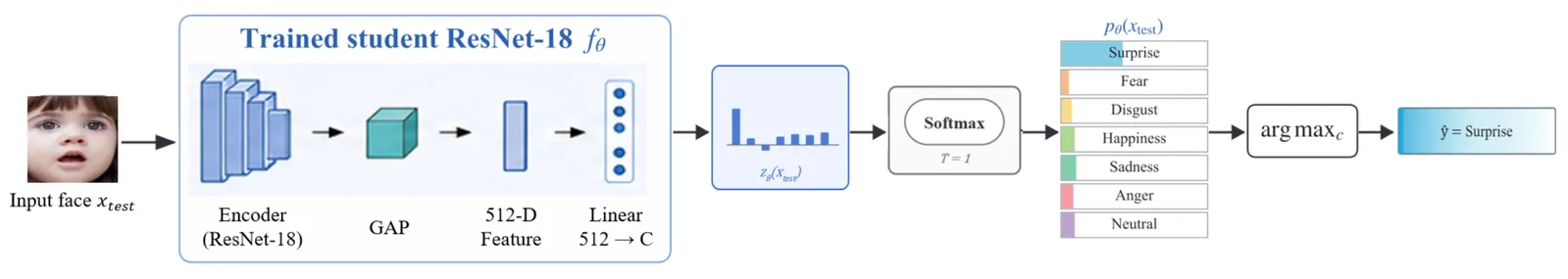
Student-only inference, illustrated schematically with an example face from the RAF-DB training split. The “input face” label in the figure identifies the input to the inference pipeline. The image passes once through ResNet-18, softmax, and $\arg\max_{c}$; HardMix, the reference teacher, paired branches, and losses are absent.

At inference, the deployment graph retains only the trained student, as illustrated in Figure 3.

For a test image $x_{\mathrm{test}}$, the retained classifier computes

(11) $$z_{\theta }\left(x_{\mathrm{test}}\right)=f_{\theta }\left(x_{\mathrm{test}}\right),\;p_{\theta }\left(x_{\mathrm{test}}\right)=\mathrm{softmax}\;\left(z_{\theta }\left(x_{\mathrm{test}}\right)\right),\;\hat{y}=\arg\max_{c\in \{1,\ldots ,C\}}p_{\theta }^{c}\left(x_{\mathrm{test}}\right).$$

Thus, deployment uses the same single-backbone graph as the baseline, without paired inputs or an occlusion-specific module.

## 4. Results

### 4.1. Experimental Setup

The evaluation protocol combines the standard seven-class RAF-DB split [38], the 734-image Occlusion-RAF-DB subset derived by the Region Attention Network (RAN) study [1], five controlled synthetic masks, AffectNet [39] and its 682-image Occl.-AffectNet subset constructed by RAN [1], and the 400-image seven-class FED-RO benchmark [21]. Table 2 summarizes dataset roles; FED-RO supports both direct transfer and five-fold adaptation.

All stages use ResNet-18 [40]. Strong Clean is initialized with weights pretrained on MS-Celeb-1M [41], and each subsequent stage starts from the final checkpoint of the preceding stage. Inputs are resized to 224×224 and normalized using ImageNet statistics. Strong augmentation comprises random resized cropping with scale (0.82,1.00) and aspect ratio (0.9,1.1), color jitter (0.15/0.15/0.10), RandAugment with two operations at magnitude 7, and random erasing with probability 0.15, area (0.02,0.12), aspect ratio (0.3,3.3), and random fill. During paired training, one random horizontal flip is shared across the two branches before branch construction; the non-HardMix and HardMix branches then receive independently sampled strong transformations.

We use AdamW with a weight decay of $10^{-4},$ a batch size of 128, 12 workers, and mixed-precision training. The learning rates in Table 1 are the initial values for their respective stages and follow cosine annealing over the complete schedule of each stage. Repeated-training results are reported as the mean and sample standard deviation across five independent runs with distinct random seeds. Seed 42 is the pre-specified primary checkpoint used in single-checkpoint figures and paired tests, and is not selected according to the observed performance. Evaluation samples and mask placements are fixed using seed 20,260,721, and final checkpoints are evaluated without test-based selection. Basic Clean is a separate diagnostic ResNet-18 trained for 20 epochs with light non-occlusion augmentation; it does not initialize any subsequent stage. Strong Clean follows the 80-epoch strong-augmentation schedule and includes low-probability random erasing. Its name indicates the absence of a paired facial occlusion branch and an occluded view loss, not that every augmented input is entirely free of pixel-level occlusion.

To determine whether robustness learned from textureless rectangular masks transfers to occluders with different appearances, we construct an additional testing-only object-overlay benchmark. It uses 25 author-owned RGBA cutouts from five categories: glasses, masks, cups, scarves, and phones. Three instances per category form a development pool used only to finalize category-level compositing rules, while two per category are held out exclusively for evaluation. None of the evaluated models was retrained, adapted, selected, or tuned using either asset pool.

For each RAF-DB test face, a fixed manifest generates one deterministic alpha-composited image per object category, yielding 15,340 paired object-occluded samples. Placement follows category-specific semantic constraints: glasses cover the eye region, masks cover the nose and mouth, cups occlude the mouth and subnasal region, scarves are placed from the lower image boundary to cover the chin and lower mouth, and phones are positioned along a lateral image boundary to partially occlude the face. The manifest fixes the source face, asset, position, scale, rotation, horizontal flip, alpha value, and category-specific shape adjustment so that every checkpoint receives pixel-identical inputs.

The categories are not matched for occluded area, opacity, or placement severity. Category-wise differences are descriptive rather than isolated appearance effects. These composites are controlled synthetic occlusions, not naturally captured occlusions; Occlusion-RAF-DB, Occl.-AffectNet, and FED-RO provide the natural-occlusion evaluations.

The local training cost and inference profiles described later were measured using an NVIDIA GeForce RTX 4060 Laptop GPU (8 GB; NVIDIA Corporation, Santa Clara, CA, USA) and an AMD Ryzen 7 7745HX CPU (Advanced Micro Devices, Inc., Santa Clara, CA, USA), running Windows 10 with Python 3.8.20, PyTorch 2.4.1+cu118, and CUDA 11.8. These hardware-specific measurements support relative comparisons of stage costs but do not establish a general training efficiency advantage.

We fixed the stage schedule as a design heuristic before reporting results on the evaluation sets; it was not selected through a benchmark-driven grid search. Table 1 lists the resulting settings. Clean-view classification remains active throughout the staged schedule. Occlusion exposure increases during the middle phase and decreases near the end, the teacher weights remain fixed, T=2 provides moderate softening, and the learning rates decrease across stages. Results from the held-out RAF-DB images, synthetic masks, Occlusion-RAF-DB, Occl.-AffectNet, and FED-RO were not used to select checkpoints or hyperparameters. The HardMix probabilities encode facial priors rather than frequencies estimated from the evaluation benchmarks. Because we performed the local robustness analysis only after fixing the operating point, that analysis does not establish global optimality.

**AffectNet-8.** All strategies use the Table 1 settings in the eight-class space. The 4000-image validation and 682-image Occl.-AffectNet sets are evaluation-only. The results reported below use one fixed final checkpoint per strategy, and as such do not estimate training-seed variability.

**FED-RO.** Direct transfer evaluates the final RAF-DB checkpoints without target-domain training. The separate class-stratified five-fold adaptation protocol (seed 42) uses sample-level rather than subject-independent splits. Subject identities are unavailable under the protocol used here; therefore, the folds cannot guarantee identity separation, and images of the same person could appear in both the training and test partitions. The adapted results consequently assess sample-level target domain adaptation, and should not be interpreted as subject-independent generalization. Within each class, samples are shuffled using seed 42 and assigned to the five folds in round-robin order, yielding 317–323 training images and 77–83 test images per fold. Each initialization is fine-tuned for 20 epochs using AdamW, a batch size of 32, a learning rate of $10^{-5},$ weight decay of $10^{-4},$ strong augmentation, label smoothing of 0.05, and no paired-occlusion loss. We evaluate the fixed final epoch without validation- or test-based checkpoint selection, and report target-label adaptation separately from direct transfer.

For the synthetic tests, we prioritize absolute accuracy and use clean-to-occlusion differences as supporting diagnostics. For the imbalanced natural occlusion sets, we report both accuracy and macro-F1, where macro-F1 is the unweighted mean of the class-wise F1 scores. Throughout the experiments, “clean” denotes an unmodified benchmark image to which no additional synthetic occluder has been applied; the term does not imply that every in-the-wild image is physically free of natural occlusion. Direct transfer to FED-RO is reported separately from label-supervised adaptation.

### 4.2. Comparison with Existing Methods

To provide literature-level context, Table 3 includes methods that report results on at least one shared natural-occlusion benchmark. We omit clean-only FER methods because they do not directly evaluate the central robustness question considered here. This inclusion rule is based on the evaluation protocol, not on performance. Table 4 and the component study reported later instead provide the controlled evidence for method attribution. Because the published methods do not share a common training and evaluation protocol, Table 3 cannot support conclusions about causal method effects, statistical superiority, or normalized computational efficiency.

**Table 3:** Source-reported classification accuracy (%) for representative methods for occlusion-robust FER, with the published methods ordered chronologically. Each included method reports results on at least one of Occlusion-RAF-DB, Occl.-AffectNet, or FED-RO; clean-only comparisons are excluded. Published values are reproduced from the source papers. The CA-HOFT row reports a single pre-specified primary checkpoint using seed 42; its five-run mean ± sample standard deviation is reported in Table 4 and Table 5 and in the controlled component analyses reported later. “Aff. protocol” denotes the number of AffectNet classes. Differences in training data, valid image lists, labels, preprocessing, and evaluation procedures mean that this table is contextual rather than a controlled ranking. A dash indicates that a result was not reported.

Method	RAF-DB Acc. (%)	Occlusion-RAF-DB Acc. (%)	FED-RO Acc. (%)	Aff. Protocol	AffectNet Acc. (%)	Occl.-AffectNet Acc. (%)
RAN [1]	86.90	82.72 ^‡^	67.98 ^†^	8	59.50	58.50
OADN [2]	89.83	–	71.17 ^†^	7	64.06 *	64.02 *
Wang et al. (self-supervised) [23]	85.95	82.45 ^‡^	70.00 ^†^	7/8	60.20 *	59.30 ^‡^
Latent-OFER [3]	89.60	84.20 ^‡^	71.80 ^†^	7	63.90 *	66.10 *
ORSANet [4]	92.28	87.75 ^‡^	–	8	62.95	–
**CA-HOFT (Ours; primary checkpoint)**	90.16	87.47	68.25	8	64.35	63.78

FER denotes facial expression recognition; RAN denotes Region Attention Network; and OADN denotes Occlusion-Adaptive Deep Network. * The cited AffectNet values use either seven classes or incompletely documented handling of the contempt class, and are provided for context; Wang et al. reported a separate eight-class result for Occl.-AffectNet. ^†^ Published FED-RO protocols differ in source data, access to target labels, preprocessing, or data splits, and are not controlled against our RAF-DB-only direct-transfer protocol, as detailed in the FED-RO evaluation below. ^‡^ The source papers do not document subset counts or valid image lists that are demonstrably identical to our fixed 734-image Occlusion-RAF-DB and 682-image Occl.-AffectNet sets.

**Table 4:** Controlled comparison on RAF-DB. Within each seed, the 24-epoch variants share the seed-matched mixed FT initialization, ResNet-18 architecture, optimizer, data split, checkpoint rule, and evaluation sets. The comparison is matched by epoch count rather than computation; equal epoch counts do not imply equal training cost, and measured stage costs are reported separately. Values are the mean ± sample standard deviation across five runs, including the pre-specified primary checkpoint using seed 42. The five-run summaries provide descriptive estimates of retraining variability rather than formal significance tests across seeds. Mean Syn. Acc. is averaged across five masks. Bold and underlined values denote the numerically highest and second-highest means, respectively.

Method	Clean Acc.	Mean Syn. Acc.	Mask-Like Acc.	Occlusion-RAF-DB Acc.	Occlusion-RAF-DB F1
Mixed FT ^†^	89.47 ± 0.16	81.62 ± 0.31	76.11 ± 0.25	86.35 ± 0.31	81.89 ± 0.24
Random Erasing FT	89.27 ± 0.23	80.64 ± 0.31	74.23 ± 0.27	85.83 ± 0.32	81.23 ± 0.28
CutMix FT	89.43 ± 0.23	80.86 ± 0.28	74.61 ± 0.36	86.13 ± 0.30	81.41 ± 0.37
Standard clean-view KD FT	89.65 ± 0.19	81.47 ± 0.28	76.44 ± 0.24	86.65 ± 0.29	82.33 ± 0.29
**CA-HOFT**	**90.08 ± 0.21**	**82.45 ± 0.26**	**77.41 ± 0.28**	**87.38 ± 0.12**	**83.34 ± 0.32**

Acc. denotes accuracy; Syn. denotes synthetic; FT denotes fine-tuning; and KD denotes knowledge distillation. ^†^ Mixed FT is the initialization stage, not an additional 24-epoch run. Random erasing FT and CutMix FT use paired classification branches without teacher losses. Standard clean-view KD uses a single view and combines clean-label cross-entropy with distillation from the fixed mixed FT reference teacher; it does not use HardMix or occluded-view classification. By contrast, the clean-view teacher-only ablation in the component study reported below retains paired non-HardMix/HardMix branches and the occluded-view loss while setting the cross-view teacher weight to zero.

**Table 5:** Testing-only evaluation using held-out alpha-composited object occlusions. All rows use the same 3068 RAF-DB test faces, deterministic manifest, and two held-out object assets per category; none of the models is retrained or adapted using these composites. The categories are not matched for occluded area, opacity, or placement severity, so category-wise differences are descriptive. “Clean” refers to the original test images without an object overlay; Object Mean Acc. is the unweighted mean accuracy across the five object categories; and Object Mean Macro-F1 is the corresponding unweighted mean of the category-wise macro-F1 scores.

Method	Clean	Glasses	Mask	Cup	Scarf	Phone	Object Mean Acc.	Object Mean Macro-F1
Strong Clean	89.29 ± 0.14	86.11 ± 0.15	64.55 ± 0.19	83.11 ± 0.14	65.93 ± 0.12	86.86 ± 0.17	77.31 ± 0.12	68.37 ± 0.11
Mixed FT	89.47 ± 0.16	87.80 ± 0.12	68.25 ± 0.17	85.61 ± 0.12	70.72 ± 0.10	88.02 ± 0.11	80.08 ± 0.13	71.91 ± 0.18
HardMix w/o teacher	89.21 ± 0.16	87.86 ± 0.13	68.35 ± 0.17	85.58 ± 0.10	70.24 ± 0.11	88.39 ± 0.14	80.08 ± 0.11	71.82 ± 0.10
CA-HOFT	90.08 ± 0.21	88.49 ± 0.21	69.40 ± 0.16	86.32 ± 0.17	72.82 ± 0.13	88.93 ± 0.11	81.19 ± 0.15	72.88 ± 0.14

Values are percentages reported as the mean ± sample standard deviation across five independently trained models. Every model is evaluated using the same deterministic held-out manifest. Each object category contains 3068 samples, yielding 15,340 object-occluded images in total. The five-run summaries are descriptive and do not constitute significance tests across training seeds. FT denotes fine-tuning.

Table 3 reports the pre-specified seed-42 primary checkpoint rather than the five-run mean: 90.16% clean accuracy and 87.47% Occlusion-RAF-DB accuracy with 83.35% macro-F1. The five-run means (90.08%, 87.38%, and 83.34%) are reserved for controlled repeated-training analyses, and are not interchangeable with this fixed checkpoint.

ORSANet has the highest source-reported RAF-DB and Occlusion-RAF-DB accuracy results (92.28% and 87.75%), exceeding the CA-HOFT primary checkpoint by 2.12 and 0.28 percentage points, respectively. However, the protocols, data lists, preprocessing, and parameter definitions are heterogeneous. The comparison also reflects different design points: ORSANet uses semantic- and landmark-guided occlusion modeling, whereas CA-HOFT confines occlusion-specific operations to training and retains a standard ResNet-18 inference graph. Thus, we use this comparison only to characterize these design choices. The 68.25% FED-RO value is direct transfer, and the 64.35%/63.78% AffectNet values are fixed-checkpoint results; controlled repeated-training baselines remain the primary evidence for attribution.

CA-HOFT follows the eight-class AffectNet protocol, while several of the cited methods either use seven classes or do not fully document their class convention. Thus, the AffectNet results in Table 3 are presented only for context, and should not be interpreted as a direct cross-method ranking.

### 4.3. Controlled Comparison of Training Strategies

All additional 24-epoch variants in Table 4 use the seed-matched mixed FT initialization and share the same ResNet-18 architecture, optimizer, data split, and checkpoint rule. Mixed FT serves as the initialization reference rather than as another 24-epoch run. The comparison is matched by epoch count, not by computation. Thus, an equal 24-epoch schedule does not imply equal training cost; CA-HOFT evaluates two student views and one teacher view per batch, while the comparator graphs use different numbers and types of forward passes.

In the same local environment, the measured CA-HOFT stage requires 4.63 min per epoch, compared with 2.85 min for standard clean-view KD. Corresponding profiles were not collected for every comparator. Table 4 supports a schedule-controlled comparison, but cannot isolate the training objective from differences in computation.

Random erasing FT and CutMix FT retain the paired branches and occluded-view loss but omit both teacher losses; their second branches use random-fill erasing or standard batch-paired CutMix, respectively.

Standard clean-view KD FT uses one view, the fixed mixed FT teacher, clean-label CE, $\lambda_{\mathrm{ct}}=0.60$, and T=2, without HardMix, occluded-view classification, or a cross-view term. The clean-view teacher-only ablation retains the paired branches and occluded-view loss. CA-HOFT has the highest five-run mean for all five endpoints. Relative to mixed FT, it improves clean accuracy by 0.61 points, mask-like accuracy by 1.30 points, natural-occlusion accuracy by 1.03 points, and natural-occlusion macro-F1 by 1.45 points. The means and standard deviations summarize retraining variability, and the rankings are descriptive; the fixed-checkpoint profile shown later is not interchangeable with these means.

### 4.4. Held-Out Alpha-Composited Object Occlusion

**Figure 4:**
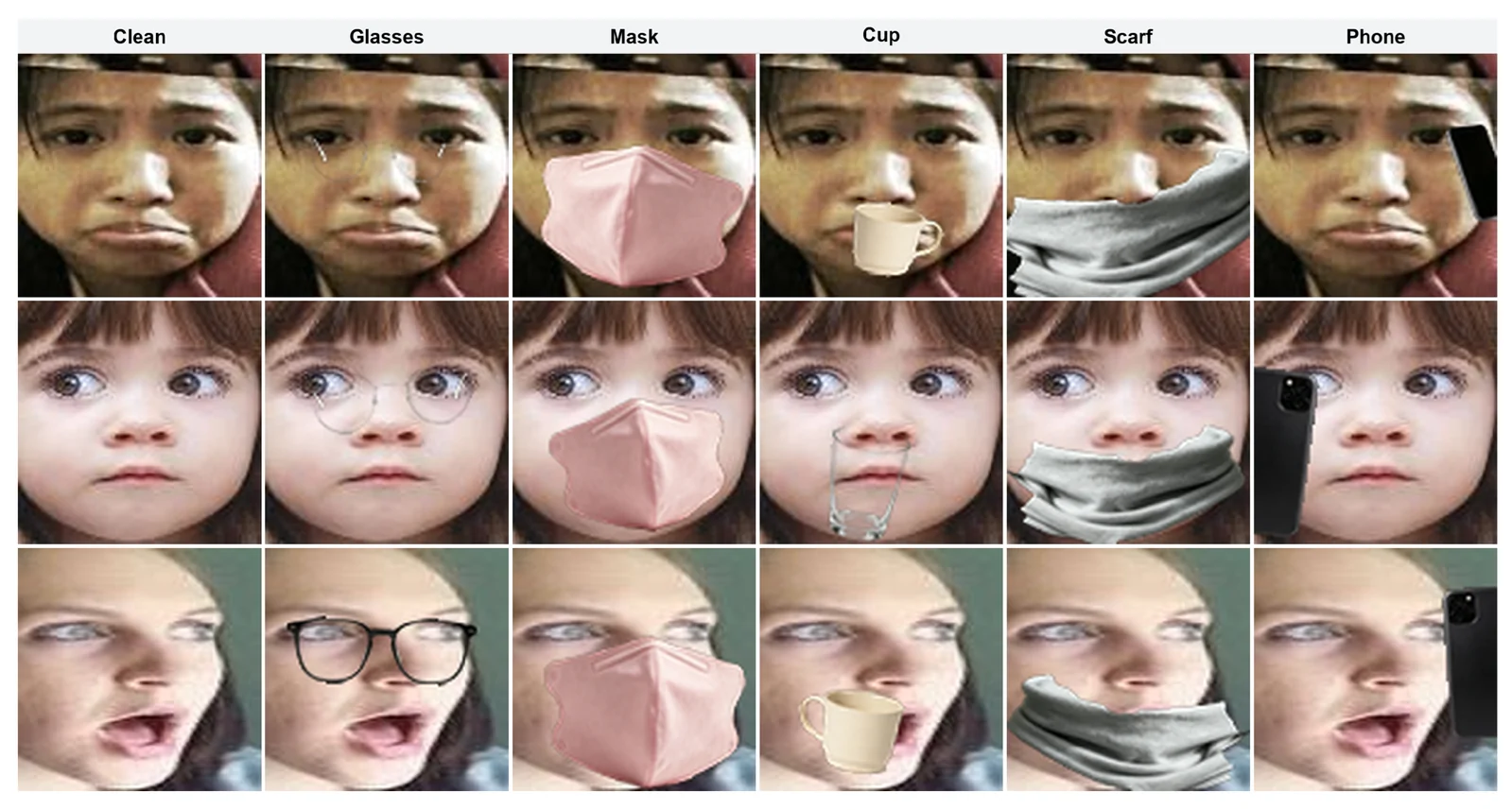
Representative clean faces and alpha-composited object-occluded variants from the testing-only benchmark. Each row shows one RAF-DB test face under five semantically placed object categories generated by the same held-out manifest used for quantitative evaluation. These examples are controlled synthetic occlusions.

The original HardMix intervention deliberately represents missing evidence using textureless rectangular masks. The present stress test examines whether the resulting robustness is confined to this training appearance. It changes the shape, texture, color, and boundary of the occluder while keeping all model parameters fixed. Figure 4 shows representative outputs generated from the same held-out object occlusion manifest used for the quantitative evaluation. We select the examples only for visual clarity, and do not reposition or rescale the objects specifically for the figure.

Table 5 evaluates Strong Clean, mixed FT, teacher-free HardMix, and CA-HOFT across five independently trained models on the same deterministic manifest without retraining or adaptation. The primary comparison evaluates each occlusion-aware strategy against Strong Clean to determine whether robustness transfers beyond the black rectangular training appearance. Comparisons among mixed FT, teacher-free HardMix, and CA-HOFT address the narrower question of whether the complete teacher-guided objective adds value under the same held-out occluder appearances, and should not be conflated with the cross-appearance test itself.

Every occlusion-aware strategy achieves a higher five-run mean than Strong Clean in each object category. CA-HOFT increases mean object accuracy from 77.31% to 81.19% and mean object macro-F1 from 68.37% to 72.88%, gains of 3.88 and 4.51 points. The largest category gains occur for masks and scarves, but the pattern is descriptive and does not establish a causal region-specific effect.

CA-HOFT has the highest five-run mean in every column. Relative to mixed FT and teacher-free HardMix, it improves mean object accuracy by 1.11 points; the corresponding macro-F1 margins are 0.97 and 1.06 points. These differences support the complete objective within the tested variants, although the held-out alpha composites are controlled synthetic occlusions that should be used to complement evaluation on natural occlusions.

**Figure 5:**
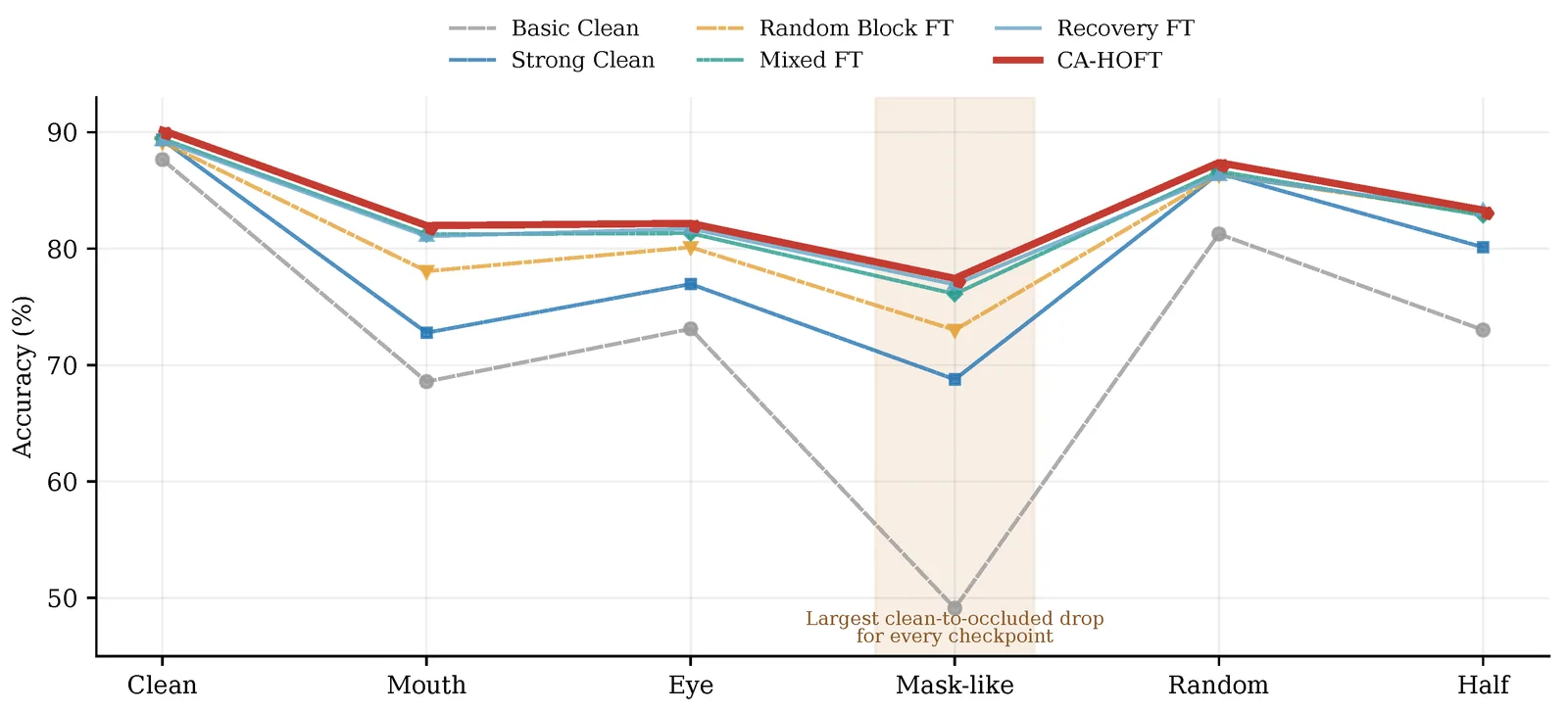
Accuracy of the pre-specified primary checkpoints on the Real-World Affective Faces Database (RAF-DB) and under five fixed synthetic masks (3068 images per setting). Basic Clean is diagnostic, whereas Strong Clean initializes mixed-occlusion fine-tuning (mixed FT). The pale band highlights the mask-like condition, which causes the largest degradation. The vertical axis is truncated to make differences within each profile legible.

The fixed-checkpoint profile shown in Figure 5 is descriptive and is not interchangeable with the five-run means.

#### Severity-Controlled Analysis

We define severity as the rectangular-mask area ratio on the same 3068 test images:

(12) $$r=\frac{|M_{r}|}{HW},\;r\in \left\{0.10,0.20,0.30,0.40,0.50\right\}.$$

Random blocks are deterministic. Mouth, eye, and mask-like rectangles expand vertically, and may include neighboring regions at high severity. All methods are evaluated using identical masks.

The severity sweep uses four role-based comparators: Strong Clean, generic random block FT, mixed FT, and CA-HOFT. Basic Clean and recovery FT are omitted. The ratio is computed from the realized in-image mask after boundary handling, using one deterministic manifest for every checkpoint.

**Figure 6:**
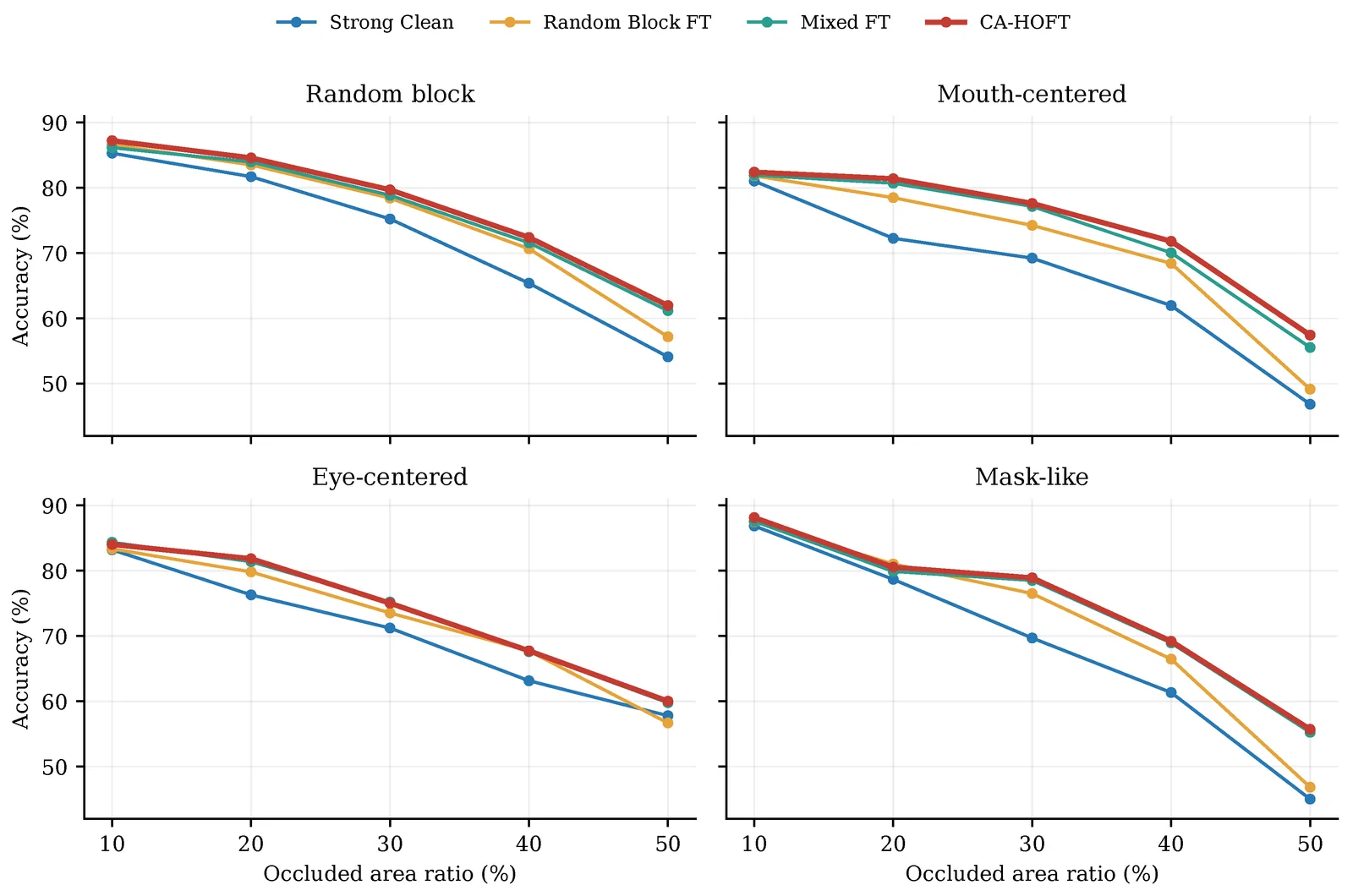
Accuracy of the pre-specified seed-42 primary checkpoints as the rectangular mask area increases on the same 3068 RAF-DB images. At high severity, semantic masks expand into neighboring regions. The shared vertical axes are truncated to emphasize degradation trends rather than absolute effect sizes.

As shown in Figure 6, accuracy decreases with severity. Across the 20 conditions, CA-HOFT averages 74.88%, versus 74.28% for mixed FT, 72.40% for random block FT, and 69.31% for Strong Clean. At the 50% area ratio, the CA-HOFT advantage over Strong Clean is 10.59 points for mouth occlusion, 10.69 points for mask-like occlusion, 7.85 points for random blocks, and 2.22 points for eye occlusion. These differences are descriptive and do not establish a causal region-specific effect.

CA-HOFT has the highest average across conditions, although individual settings favor other models; every model declines at high severity, consistent with the inability of consistency regularization alone to recover visual evidence removed from the input.

### 4.5. Evaluation on Additional Datasets and Natural Occlusion

#### 4.5.1. AffectNet-8 and Occl.-AffectNet

The single-checkpoint AffectNet-8 evaluation follows the Strong Clean–mixed FT–CA-HOFT progression. Table 6 reports the results. CA-HOFT achieves 64.35% clean accuracy, 58.75% mean synthetic accuracy, 63.78% Occl.-AffectNet accuracy, and 63.15% macro-F1.

Recovery FT exceeds CA-HOFT by 0.06 points in mean synthetic accuracy, whereas CA-HOFT has higher clean and natural-occlusion point estimates. Only the CA-HOFT versus Strong Clean accuracy comparison remains significant after Holm correction; no macro-F1 comparison remains significant. This single-checkpoint evaluation does not establish seed-level consistency.

#### 4.5.2. FED-RO

On FED-RO, CA-HOFT achieves 68.25% accuracy and 64.18% macro-F1 under direct transfer, with higher point estimates than the corresponding Strong Clean and mixed FT baselines. However, the cross-paper protocol differences summarized in Table 3 still apply. Five-fold supervised adaptation yields 69.30% accuracy and 67.08% macro-F1. Its margins over recovery FT are 0.52 and 0.51 percentage points for accuracy and macro-F1, respectively. Because these differences are substantially smaller than the fold-to-fold variability, we do not interpret them as evidence of statistical superiority.

Every supervised fold uses the same target-domain objective without a paired-occlusion loss, so this experiment evaluates transfer quality rather than the complete CA-HOFT objective. The class-stratified folds are sample-level, and are not verified to be subject-independent because identity labels are unavailable; therefore, the adapted results support sample-level target domain adaptation only.

Table 7 summarizes the direct-transfer and supervised-adaptation results.

For the paired analysis in Table 8, the role-based comparators are Strong Clean, the mixed FT initialization, standard clean-view KD, teacher-free HardMix, and optional recovery FT. The AffectNet panel contains the corresponding available checkpoints. Recovery FT was included in the Holm correction family whenever it was reported within a benchmark–endpoint analysis, ensuring consistent family-wise error control across all reported CA-HOFT pairwise comparisons. Its inclusion increases the number of hypotheses and results in a more conservative multiplicity adjustment.

The analysis uses 10,000 paired bootstrap resamples. Accuracy is evaluated with exact two-sided McNemar tests, whereas macro-F1 uses class-stratified bootstrap resampling. Holm correction is applied within each benchmark and endpoint at α=0.05. Pointwise 95% confidence intervals are defined by the empirical 2.5th and 97.5th percentiles of the corresponding bootstrap distribution. The macro-F1 tail probability equals twice the smaller smoothed resampled proportion on either side of zero. The intervals are unadjusted, whereas the displayed *p*-values are Holm-adjusted; therefore, inferential conclusions follow the adjusted tests. None of the Occlusion-RAF-DB comparisons remains significant. On Occl.-AffectNet, only the accuracy comparison with Strong Clean is significant ($p_{\mathrm{Holm}}=0.034$). These tests quantify uncertainty across evaluation samples for fixed checkpoints, not uncertainty arising from retraining.

**Figure 7:**
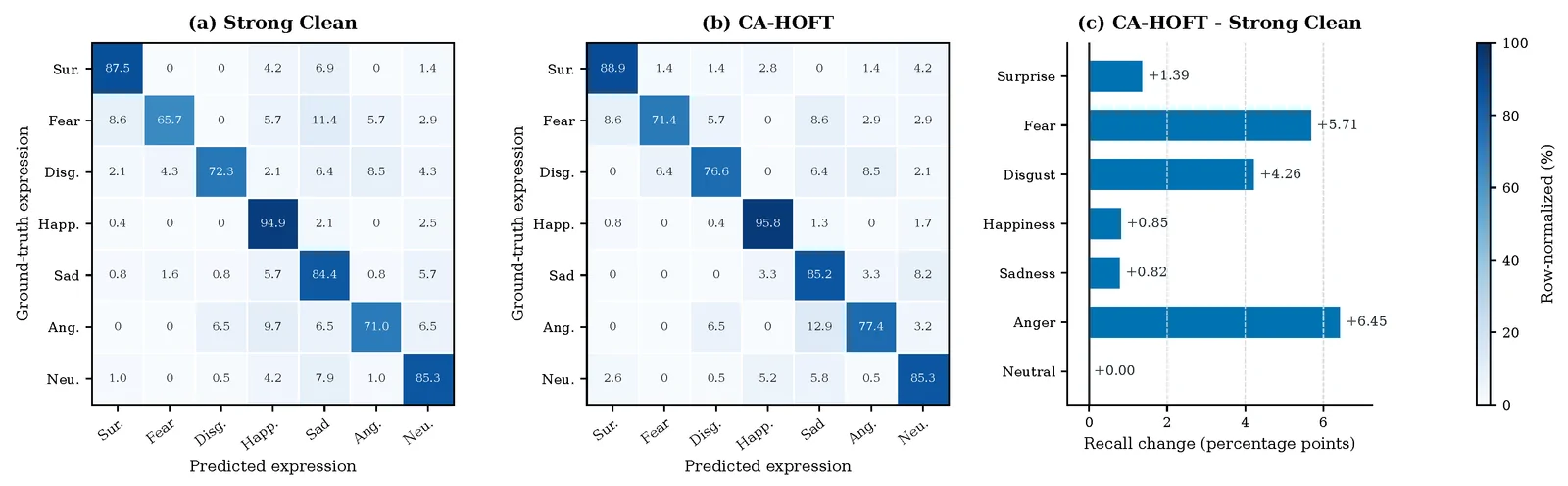
Row-normalized Occlusion-RAF-DB confusion matrices for the primary Strong Clean and CA-HOFT checkpoints, with CA-HOFT minus Strong Clean recall changes. Counts reproduce 632/734 versus 642/734 correct predictions; class-level changes are descriptive.

At the primary checkpoints, Strong Clean correctly classifies 632 of 734 images and achieves 81.82% macro-F1, whereas CA-HOFT correctly classifies 642 images and achieves 83.35% macro-F1 (Figure 7). Recall improves most for anger, fear, and disgust while remaining unchanged for the neutral class, suggesting that the gains are not confined to the majority class. Residual errors are concentrated between sadness and neutral, with additional confusion from neutral to happiness. These visually similar class pairs remain challenging. All class-level changes are descriptive.

**Figure 8:**
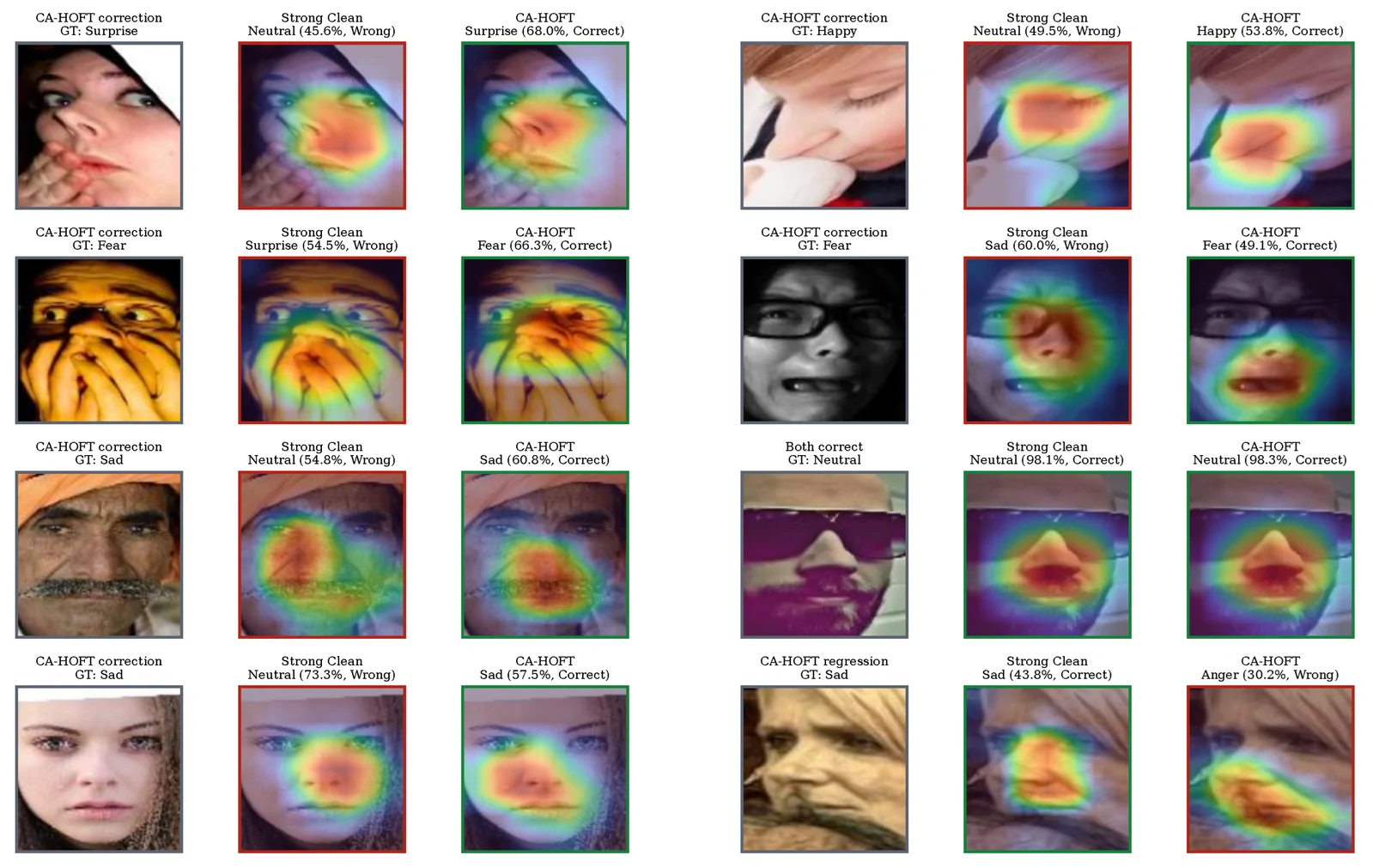
Eight illustrative Occlusion-RAF-DB Grad-CAM cases selected by prediction outcome: six CA-HOFT corrections, one shared correct prediction, and one CA-HOFT regression. Each triplet contains the unmodified benchmark image, Strong Clean map, and CA-HOFT map. Maps target each model’s predicted class and are normalized independently; within each heatmap, warmer red–yellow regions indicate relatively stronger activation and cooler green–blue regions indicate weaker activation. Color intensity is not quantitatively comparable across panels or examples; green/red borders denote correct/incorrect predictions.

Figure 8 presents eight outcome-selected Occlusion-RAF-DB examples: six corrections, one shared correct prediction, and one CA-HOFT regression. The first image in each triplet is the unmodified benchmark input rather than a clean-control condition; because the cases are selected by prediction outcome, the obstruction can be localized or visually inconspicuous. The cases are qualitative; dataset-level conclusions rely on the paired tests and confusion matrices.

### 4.6. Ablation and Sensitivity Analysis

The component study in Table 9 keeps the deployed architecture fixed. Panel (a) compares the staged and HardMix variants; panel (b) varies teacher terms while retaining clean-label classification. The matched comparison between “HardMix w/o clean anchor” in panel (a) and “cross-view teacher only” in panel (b) isolates the contribution of clean-label cross-entropy while retaining the cross-view teacher term. Both panels report five-run means and sample standard deviations, which are interpreted descriptively.

The table separates synthetic robustness from natural-occlusion transfer. Teacher-free HardMix performs worse than mixed FT on every evaluated endpoint, whereas adding both teacher terms improves all endpoints. Removing the bundled clean anchor also reduces performance, but this row cannot attribute the change to clean-label CE or clean-view consistency separately.

The matched contrast gives small clean-label CE gains: adding the clean-label term increases clean accuracy by 0.38 points, mask-like accuracy by 0.06 points, natural-occlusion accuracy by 1.03 points, and natural-occlusion macro-F1 by 0.68 points. Each teacher term individually improves upon teacher-free HardMix, and their joint use gives the highest mean in every column. Table 9 is not a full factorial design; only the matched contrast isolates clean-label CE, and the remaining comparisons are descriptive rather than independent causal analyses or tests of statistical significance.

#### Sensitivity Analysis

Table 10 evaluates local sensitivity around the pre-specified operating point. All variants share the seed-matched mixed FT initialization, ResNet-18 backbone, optimizer, 24-epoch schedule, checkpoint rule, and fixed evaluation sets.

This experiment assesses local robustness rather than selecting parameters. The default teacher weights give the highest mean for four of five metrics; increasing the clean-teacher weight raises mean synthetic accuracy by only 0.04 points while lowering natural-occlusion accuracy by 1.30 points. A plausible but unverified interpretation is that a larger clean-teacher weight constrains the student more tightly to the fixed mixed FT reference, which may preserve the controlled synthetic mask profile while reducing flexibility under natural appearance shifts that are not fully represented by that reference. This remains a hypothesis rather than a causal finding. Changing either teacher weight or the HardMix schedule produces local trade-offs, and replacing the facial prior with a uniform distribution lowers all five means. The tested neighborhood favors the default joint weighting, but does not establish a global optimum or a formal significance result.

Table 10b separates the activation schedule from the distribution of occlusion type. Lower or higher schedules trade clean accuracy against robustness, while a uniform distribution lowers all five means. This comparison is descriptive only.

**Figure 9:**
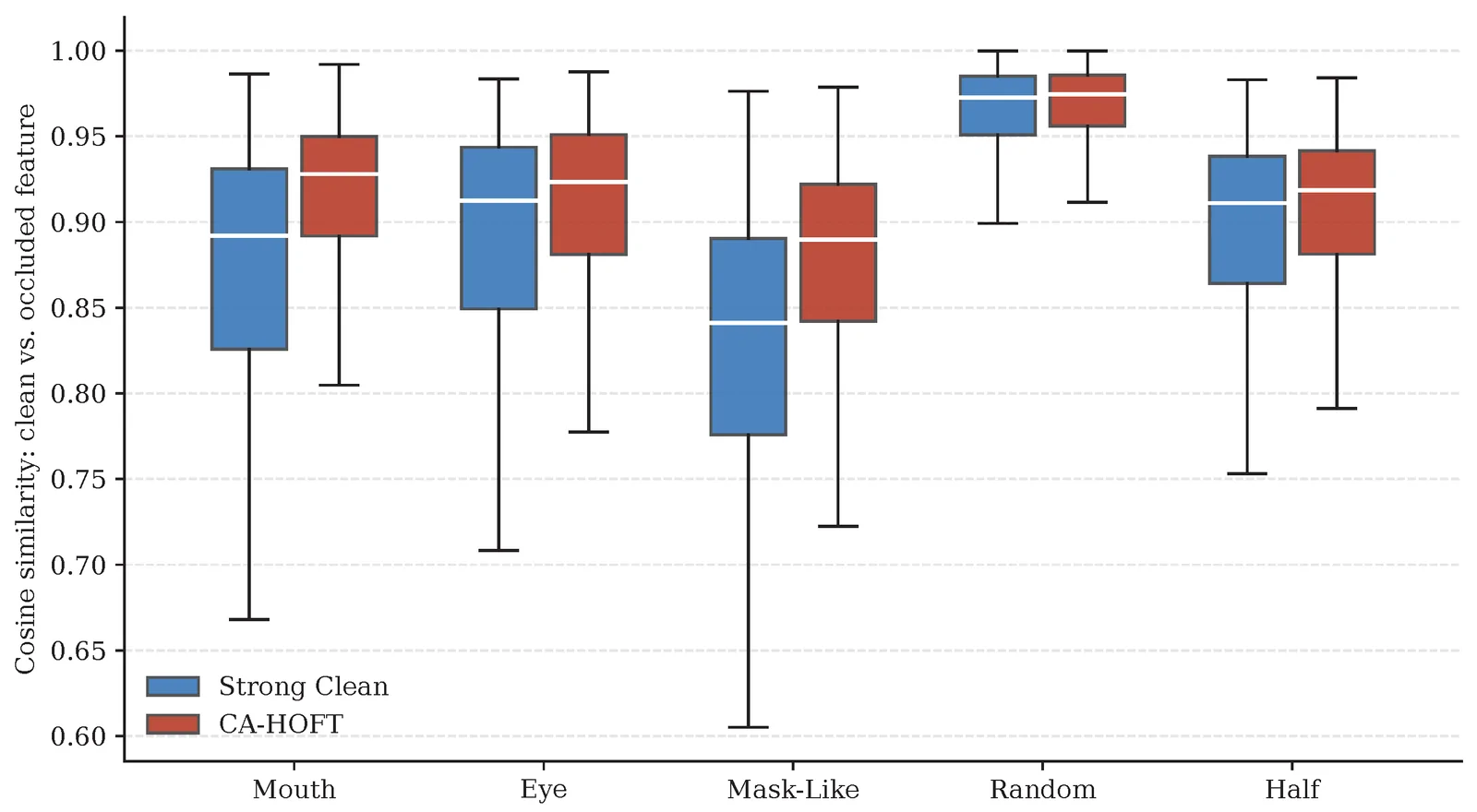
Feature consistency under synthetic occlusion in the original representation space. Cosine similarity is computed between $l_{2}$-normalized, 512-dimensional global average pooled features for the clean and occluded versions of all 3068 RAF-DB test images under five fixed occlusions (15,340 pairs per model). The boxes show the interquartile range. Higher similarity indicates less drift between paired views. Both models use their pre-specified seed-42 primary checkpoints and are evaluated with identical masks.

We also measure paired-view representation drift directly in the original 512-dimensional feature space (Figure 9).

Across the complete paired set, CA-HOFT has a higher median cosine similarity under all five occlusion types, with the largest visual separation under mask-like and mouth occlusion. This pattern is consistent with less drift between paired views in the original feature space. We interpret this only as behavioral support for the proposed anchoring mechanism, not as an independent measure of classification quality.

### 4.7. Computational Cost and Practical Inference

At deployment, CA-HOFT retains a single 11.18M-parameter ResNet-18 requiring 1.814 GMACs, without any reconstruction, landmark, or semantic prior pipeline. With a batch size of one, 200 warm-up iterations, and five repeats of 1000 timed iterations, 32-bit floating-point (FP32) inference takes 3.177±0.070 ms on the RTX 4060 Laptop GPU. The corresponding 95th-percentile latency (P95) is 5.119 ms and the throughput is 314.73 frames per second (FPS). On the eight-thread Ryzen 7 7745HX CPU, inference takes 14.308±0.858 ms with a P95 of 15.649 ms and a throughput of 69.89 FPS.

Here, “compact” describes the retained recognition graph and its deployment cost; it does not imply that ResNet-18 is the smallest available FER backbone or that the training procedure is computationally inexpensive. Table 11 reports the one-time local training overhead and the deployment performance of the retained model. Table 12 places its inference complexity and natural-occlusion accuracy within the context of the previous literature.

Relative to Strong Clean, CA-HOFT requires 2.24× as much epoch time and 1.71× as much allocated memory. Relative to mixed FT, the corresponding increases are 26.8% and 26.0%. The required Strong Clean–Mixed FT–CA-HOFT sequence takes 6.77 h in the local environment, or 7.78 h when the optional recovery FT stage is included. These values represent one-time training overhead, not a training efficiency advantage. Table 11b measures network-only inference and excludes image decoding, face detection, preprocessing, and host-to-device transfer.

Under the heterogeneous definitions summarized in Table 12, the CA-HOFT deployment graph is an 11.18M-parameter ResNet-18 and its Occlusion-RAF-DB accuracy is 0.28 percentage points below that of ORSANet, the highest source-reported entry. The parameter counts are not directly comparable. ORSANet reports only its trainable portion, CA-HOFT reports the complete retained ResNet-18 graph, and the other sources apply their own counting conventions; accordingly, “compact” refers to CA-HOFT’s retained single-backbone inference graph and measured local deployment cost, but does not imply that CA-HOFT has been established as the smallest or most efficient method in a normalized cross-paper comparison.

**Figure 10:**
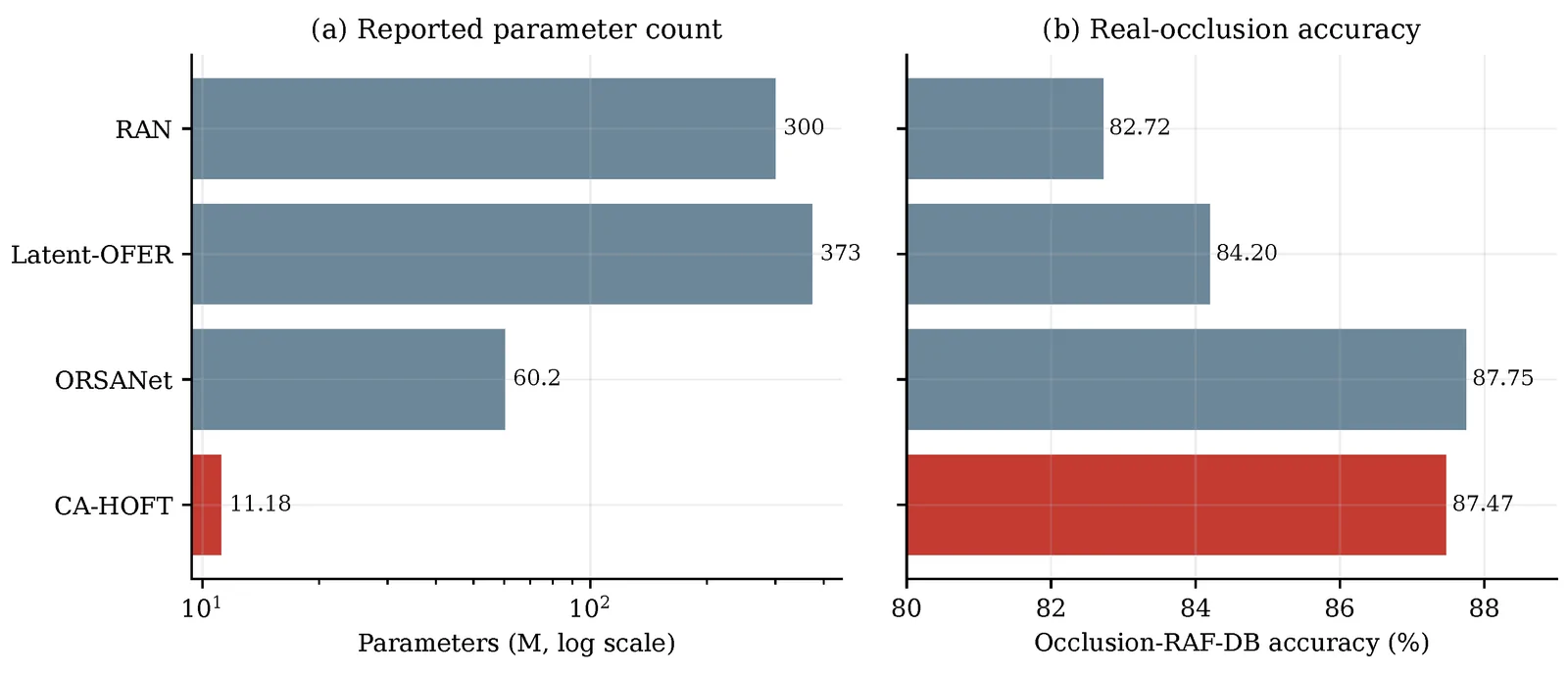
Literature-level summary of reported model size and Occlusion-RAF-DB accuracy for the four methods in Table 12. Panel (**a**) uses a logarithmic parameter axis and each source’s own parameter definition; panel (**b**) uses a restricted accuracy range to make small differences legible. Clean-anchored hard occlusion fine-tuning (CA-HOFT) is highlighted in red for identification. Region Attention Network (RAN) complexity is reproduced from the unified estimate in Table 7 of Latent-OFER. Latent-OFER reports its own complexity, ORSANet reports only its trainable portion, and CA-HOFT uses the measured deployed ResNet-18 parameter count. Interpretation follows the heterogeneous source definitions and limitations stated in Table 12.

The same source-reported relationship is visualized in Figure 10 under the heterogeneous definitions summarized in Table 12; CA-HOFT is highlighted only for identification.

## 5. Discussion

The central question addressed by this paper is whether hard-occlusion learning can improve tolerance to missing evidence without sacrificing absolute clean-image performance. Under the evaluated RAF-DB protocol, CA-HOFT provides the most favorable observed cleanness–robustness trade-off among the tested epoch-matched final-stage alternatives. This comparison is not compute-matched, and independently augmented branches make the cross-view term a combined view-change constraint rather than an occlusion-only intervention. The ablations support the roles of clean-label supervision and reference transfer, but the bundled comparisons are descriptive and do not estimate all interactions or establish universal statistical superiority. Recovery FT further shows that a smaller synthetic drop does not guarantee the best absolute clean or natural-occlusion performance.

The matched comparison between the “HardMix w/o clean anchor” variant in panel (a) and the “cross-view teacher only” variant in panel (b) is the most interpretable component comparison: it isolates the clean-label CE term while retaining the cross-view teacher term. The remaining ablation rows change more than one factor, and as such show compatibility with the proposed design rather than independent causal effects. The sensitivity study provides local support for the selected operating point within the tested hyperparameter neighborhood. Together, these analyses connect the observed performance pattern to the intended clean anchor and reference transfer roles without overstating attribution.

The testing-only object overlay evaluation using held-out occluder assets provides descriptive evidence that the benefit is not confined to textureless rectangular training masks: CA-HOFT reaches 81.19% mean object accuracy and 72.88% mean object macro-F1, exceeding Strong Clean by 3.88 and 4.51 points. None of the models used the held-out assets for training or selection. Because the asset set is small and categories are not matched for area, opacity, or placement severity, these controlled composites complement rather than replace naturally captured occlusions.

The severity curves and feature space analysis are consistent with improved tolerance and lower paired-view drift, but CA-HOFT does not lead at every operating point and cannot reconstruct removed evidence. Residual confusion between sadness/neutral and neutral/happiness further indicates that visually similar classes remain difficult when evidence is incomplete. The cosine analysis provides behavioral support for reduced paired-view drift, not an independent measure of class discrimination or a causal mechanism.

The paired tests and five-run summaries quantify different uncertainty sources. Paired tests characterize variation across evaluation samples for pre-specified fixed checkpoints, whereas the five independent training runs provide a descriptive estimate of retraining variability. Fixed-checkpoint tests on Occlusion-RAF-DB are not significant after Holm correction; only the Occl.-AffectNet accuracy comparison with Strong Clean remains significant, and no macro-F1 comparison remains significant. AffectNet-8 and direct or adapted FED-RO results provide supporting evidence with single-checkpoint, sample-level, and cross-domain limitations. The conclusions rely on agreement among controlled means, ablations, stress tests, and natural-occlusion results rather than universal pairwise significance.

At deployment, the retained graph is a single 11.18M-parameter, 1.814-GMAC ResNet-18; the teacher, paired branches, and HardMix generator are training-only. This avoids an occlusion-specific deployment pipeline at the cost of additional training computation. The reported latency is network-only and specific to the RTX 4060 Laptop GPU and Ryzen 7 7745HX CPU hardware. Cross-paper size and speed comparisons remain contextual because the sources used heterogeneous definitions.

Limitations include the small held-out asset set, in which categories were not matched for occluded area, opacity, or placement severity; the small size of the natural-occlusion evaluations; retraining uncertainty that was not captured by paired tests; FED-RO folds that were defined at the sample level and were not verified as subject-independent; and restriction to static-image FER with ResNet-18. The benchmark labels represent categorical expression annotations rather than direct measurements of internal affect. Future work should examine demographic and clinical settings, temporal and multimodal inputs, alternative teacher designs, and broader cross-domain validation.

## 6. Conclusions

This study has examined whether a compact FER model can learn from missing facial evidence while preserving clean-image discrimination. CA-HOFT combines HardMix weighted by facial region, clean-label supervision, and a fixed non-HardMix reference distribution within one student model. Across five independent runs, CA-HOFT achieves 90.08 ± 0.21% accuracy on RAF-DB, 87.38 ± 0.12% accuracy with 83.34 ± 0.32% macro-F1 on Occlusion-RAF-DB, and 81.19 ± 0.15% mean object accuracy with 72.88 ± 0.14% mean object macro-F1 on the testing-only object overlay evaluation using held-out occluder assets.

These results provide descriptive evidence for CA-HOFT within the evaluated settings. They should not be interpreted as compute-matched causal evidence or as evidence of universal statistical superiority. Fixed-checkpoint Occlusion-RAF-DB comparisons are not significant after Holm correction, and the evidence is limited by the controlled asset set, small natural-occlusion evaluations, sample-level FED-RO protocol, and static-image ResNet-18 setting. Nonetheless, the retained student model with 11.18M parameters and 1.814 GMACs supports single-backbone inference under the evaluated protocols.

## Figures and Tables

**Table 1:** Main RAF-DB schedule. Each final checkpoint initializes the next stage; $p_{\mathrm{occ}}$ is distinct from $q_{\mathrm{hard}}$, and all occlusion and teacher operations are training-only.

Stage	Epochs	LR	LS	Occ. Mode	Occ. Prob.	$\lambda_{\mathrm{occ}}$	$\lambda_{\mathrm{ct}}$	$\lambda_{\mathrm{ot}}$	*T*
Strong Clean	80	$2\;\times \;10^{-4}$	0.05	None	0.00	0.00	0.00	0.00	–
Mixed FT	30	$5\;\times \;10^{-5}$	0.03	Mixed	0.80	0.70	0.00	0.00	–
CA-HOFT	24	$8\;\times \;10^{-6}$	0.02	HardMix	0.40–0.65	0.40–0.80	0.60	0.25	2.0
Recovery FT	12	$3\;\times \;10^{-6}$	0.01	HardMix	0.20–0.30	0.15–0.25	0.80	0.15	2.0

RAF-DB denotes the Real-World Affective Faces Database; CA-HOFT denotes clean-anchored hard occlusion fine-tuning; LS denotes label smoothing; LR denotes the initial learning rate, which is cosine-annealed over the corresponding stage; FT denotes fine-tuning; Occ. denotes occlusion; Occ. mode and Occ. prob. refer specifically to the paired facial occlusion branch; generic random erasing remains part of the Strong Clean augmentation pipeline. For CA-HOFT, $\left(p_{\mathrm{occ}},\lambda_{\mathrm{occ}}\right)$ is (0.40,0.40) in epochs 1–4, (0.55,0.60) in epochs 5–12, (0.65,0.80) in epochs 13–20, and (0.45,0.50) in epochs 21–24. For Recovery FT it is (0.25,0.20) in epochs 1–4, (0.30,0.25) in epochs 5–8, and (0.20,0.15) in epochs 9–12. The teacher weights and temperature remain fixed within each stage.

**Table 2:** Datasets and evaluation roles. AffectNet and Occl.-AffectNet use the same eight-class label space, including contempt.

Dataset	Classes	Images Used	Occlusion Type	Role in This Study
RAF-DB	7	12,271 train/3068 test	Clean/in-the-wild	Main clean benchmark
Occlusion-RAF-DB	7	734 test	Real occlusion	Main real-occlusion benchmark
Synthetic RAF-DB	7	3068 test per setting	Mouth, eye, mask-like, random, half	Controlled robustness analysis
Object-overlay RAF-DB	7	3068 test × 5 categories	Held-out alpha- composited objects	Cross-appearance stress test
AffectNet	8	Approximately 280,000 train/ 4000 validation	Clean and synthetic occlusion	Large-scale secondary benchmark
Occl.-AffectNet	8	682 test	Real occlusion	RAN-constructed public benchmark
FED-RO	7	400	Real occlusion	Cross-dataset and supervised adaptation analysis

RAF-DB denotes the Real-World Affective Faces Database; FED-RO denotes the Facial Expression Dataset with Real-World Occlusion.

**Table 6:** Single-checkpoint AffectNet-8 evaluation using the pre-specified seed-42 primary checkpoint for each strategy. Clean accuracy and mean synthetic accuracy across five masks are evaluated on the fixed 4000-image validation split; the natural occlusion metrics use the complete 682-image Occl.-AffectNet benchmark constructed by RAN. Bold and underlined values denote the numerically highest and second-highest results, respectively, rather than statistical significance.

Model	Clean Acc.	Mean Syn. Acc.	Occl.-AffectNet Acc.	Macro-F1
Strong Clean	62.43	50.67	60.12	60.03
Mixed FT	63.18	56.22	61.44	61.13
CA-HOFT	**64.35**	58.75	**63.78**	**63.15**
Recovery FT	64.13	**58.81**	63.20	62.26

**Table 7:** FED-RO evaluation under two protocols. Direct transfer uses the pre-specified seed-42 primary RAF-DB checkpoint for each initialization without FED-RO labels, whereas supervised adaptation reports the mean ± sample standard deviation across five class-stratified sample-level folds. The two blocks are not directly comparable; the adaptation folds are defined at the sample level, and are not verified to be subject-independent. Because identity labels are unavailable under this protocol, possible identity overlap between the training and test folds cannot be excluded; therefore, the adapted results should not be interpreted as evidence of subject-independent generalization. Bold and underlined values indicate numerical rank rather than statistical significance.

Initialization	Direct Acc.	Direct F1	Adapted Acc.	Adapted F1
Strong Clean	63.00	58.11	65.76 ± 3.36	63.26 ± 3.97
Mixed FT	64.75	60.89	66.97 ± 3.88	64.23 ± 3.52
Recovery FT	67.25	63.71	68.78 ± 3.49	66.57 ± 4.16
CA-HOFT	**68.25**	**64.18**	**69.30 ± 3.44**	**67.08 ± 4.12**

**Table 8:** Paired analysis of fixed natural-occlusion predictions from the pre-specified seed-42 primary checkpoints. The primary run is not selected according to the benchmark, endpoint, or observed result. Differences are computed as CA-HOFT minus the comparator. Accuracy is analyzed using 10,000 paired-bootstrap resamples and exact two-sided McNemar tests; macro-F1 is analyzed using 10,000 class-stratified paired resamples. Holm correction is applied within each benchmark and endpoint. The statistics use unrounded predictions, evaluation seed 20,260,721, and bootstrap seed 20,260,725.

**(a) Occlusion-RAF-DB (734 images)**						
**Comparator**	**Δ** **Acc.**	**95% CI**	**McNemar** $p_{\mathrm{Holm}}$	**Δ** **F1**	**95% CI**	**Bootstrap Tail** $p_{\mathrm{Holm}}$
Strong Clean	+1.36	[+0.14,+2.59]	0.210	+1.53	[−0.7,+3.7]	0.092
Mixed FT	+1.09	[−0.14,+2.32]	0.328	+1.41	[−0.7,+3.6]	0.141
Standard clean-view KD FT	+0.82	[0.00,+1.63]	0.328	+1.00	[−1.2,+3.3]	0.147
HardMix w/o teacher	+1.77	[+0.27,+3.27]	0.175	+2.30	[−0.4,+4.8]	0.082
Recovery FT	+0.95	[−0.14,+2.18]	0.328	+1.02	[−1.1,+3.3]	0.116
**(b) Occl.-AffectNet (682 images)**						
**Comparator**	**Δ** **Acc.**	**95% CI**	**McNemar** $p_{\mathrm{Holm}}$	**Δ** **F1**	**95% CI**	**Bootstrap Tail** $p_{\mathrm{Holm}}$
Strong Clean	+3.67	[+0.88,+6.45]	0.034	+3.12	[+0.10,+6.15]	0.097
Mixed FT	+2.35	[−0.44,+5.13]	0.235	+2.02	[−1.12,+4.91]	0.285
Recovery FT	+0.59	[−0.29,+1.47]	0.235	+0.89	[−2.26,+4.13]	0.416

Differences are expressed in percentage points, and positive values favor CA-HOFT. The primary CA-HOFT reference values are: 87.47% accuracy and 83.35% macro-F1 on Occlusion-RAF-DB; 63.78% accuracy and 63.15% macro-F1 on Occl.-AffectNet. Confidence intervals are pointwise empirical percentile intervals, and are not multiplicity-adjusted; the displayed *p*-values are Holm-adjusted. These tests quantify uncertainty across evaluation samples for fixed checkpoints, not variability across repeated training runs.

**Table 9:** Controlled ablations of the training components and teacher losses. Panel (**a**) compares training variants, whereas panel (**b**) varies the teacher terms while retaining clean-label classification; “w/o clean anchor” removes clean-label cross-entropy and clean-view teacher consistency but retains cross-view consistency; Strong Clean includes low-probability random erasing; and all variants deploy ResNet-18. Values are the mean ± sample standard deviation across five runs with identical splits and evaluation sets. The clean and mask-like metrics are evaluated on RAF-DB, whereas the natural-occlusion metrics are evaluated on Occlusion-RAF-DB. The “w/o clean anchor” row represents a bundled removal rather than an independent-factor ablation. Only its matched comparison with “cross-view teacher only” in panel (**b**) isolates clean-label cross-entropy while retaining the cross-view teacher term. The table is not a full factorial design and does not estimate interactions. The five-run numerical rankings are descriptive; no formal significance tests across seeds are reported. Bold and underlined values indicate numerical rank rather than statistical significance.

(**a) Major training components**							
**Variant**	**Clean Branch**	**Occlusion**	**Teacher**	**Clean**	**Mask-like**	**Real Acc.**	**Real F1**
Strong Clean	Yes	None	No	89.29 ± 0.14	68.73 ± 0.26	86.08 ± 0.18	81.82 ± 0.29
Random Block FT	Yes	Random	No	89.02 ± 0.21	72.83 ± 0.27	85.48 ± 0.25	80.62 ± 0.27
Mixed FT	Yes	Mixed	No	89.47 ± 0.16	76.11 ± 0.25	86.35 ± 0.31	81.89 ± 0.24
HardMix w/o teacher	Yes	HardMix	No	89.21 ± 0.16	74.21 ± 0.33	85.69 ± 0.29	80.98 ± 0.29
HardMix w/o clean anchor	No	HardMix	Cross-view only	89.08 ± 0.24	76.69 ± 0.34	85.75 ± 0.21	81.43 ± 0.31
**CA-HOFT**	Yes	HardMix	Yes	**90.08 ± 0.21**	**77.41 ± 0.28**	**87.38 ± 0.12**	**83.34 ± 0.32**
**(b) Independent teacher-loss ablation**							
**Variant**		$\lambda_{\mathrm{ct}}$	$\lambda_{\mathrm{ot}}$	**Clean**	**Mask-like**	**Real Acc.**	**Real F1**
HardMix w/o teacher		0.00	0.00	89.21 ± 0.16	74.21 ± 0.33	85.69 ± 0.29	80.98 ± 0.29
Clean-view teacher only		0.60	0.00	89.57 ± 0.20	76.87 ± 0.34	86.62 ± 0.30	82.31 ± 0.35
Cross-view teacher only		0.00	0.25	89.46 ± 0.18	76.75 ± 0.37	86.78 ± 0.25	82.11 ± 0.36
**CA-HOFT (both)**		0.60	0.25	**90.08 ± 0.21**	**77.41 ± 0.28**	**87.38 ± 0.12**	**83.34 ± 0.32**

**Table 10:** Local sensitivity around the pre-specified operating point, which was fixed before this analysis and was not selected from its results. All settings share the seed-matched mixed FT initialization, ResNet-18 architecture, 24-epoch schedule, optimizer, checkpoint rule, and evaluation sets. Values are the five-run mean ± sample standard deviation; Mean Syn. is averaged across five masks. Bold and underlined values indicate numerical rank rather than statistical significance.

(**a) Teacher-weight sensitivity**							
**Setting**	$\lambda_{\mathrm{ct}}$	$\lambda_{\mathrm{ot}}$	**Clean**	**Mean Syn.**	**Mask-like**	**Real Acc.**	**Real F1**
Lower clean-teacher weight	0.30	0.25	89.38 ± 0.25	81.56 ± 0.37	76.92 ± 0.37	86.81 ± 0.22	82.63 ± 0.34
Default	0.60	0.25	**90.08 ± 0.21**	82.45 ± 0.26	**77.41 ± 0.28**	**87.38 ± 0.12**	**83.34 ± 0.32**
Higher clean-teacher weight	0.90	0.25	89.87 ± 0.27	**82.49 ± 0.31**	76.58 ± 0.36	86.08 ± 0.30	82.09 ± 0.28
Lower cross-view weight	0.60	0.10	89.70 ± 0.26	81.69 ± 0.28	76.51 ± 0.37	86.38 ± 0.35	82.05 ± 0.29
Higher cross-view weight	0.60	0.40	89.77 ± 0.24	81.96 ± 0.31	77.09 ± 0.28	86.98 ± 0.23	82.87 ± 0.35
**(b) HardMix activation and type-distribution sensitivity**							
**Activation Schedule**		**Type Distribution**	**Clean**	**Mean Syn.**	**Mask-like**	**Real Acc.**	**Real F1**
Low: 0.25/0.35/0.45/0.30		Facial prior	**90.12 ± 0.23**	81.65 ± 0.32	76.87 ± 0.29	86.84 ± 0.23	82.63 ± 0.36
Default: 0.40/0.55/0.65/0.45		Facial prior	90.08 ± 0.21	**82.45 ± 0.26**	**77.41 ± 0.28**	**87.38 ± 0.12**	**83.34 ± 0.32**
High: 0.55/0.70/0.80/0.60		Facial prior	89.56 ± 0.29	82.06 ± 0.27	77.34 ± 0.30	87.17 ± 0.24	82.89 ± 0.31
Default: 0.40/0.55/0.65/0.45		Uniform	89.78 ± 0.26	81.56 ± 0.32	76.76 ± 0.33	86.51 ± 0.29	82.62 ± 0.34

The facial prior distribution over occlusion types is (0.35,0.20,0.20,0.10,0.10,0.05) for mask-like, mouth, eye, half-face, random-block, and random-region occlusion, respectively. The uniform setting assigns a probability of 1/6 to each type.

**Table 11:** Measured local training overhead and deployment performance. Panel (**a**) reports the cost of each stage under one GPU and software configuration. Mean epoch time excludes the first epoch, whereas total stage time includes initialization, validation, and checkpointing. Panel (**b**) reports network-only inference with a batch size of one after 200 warm-up iterations and five repeats of 1000 timed iterations on the two devices in the local workstation. These measurements are hardware-specific, and are not intended to establish latency generalization across devices. The deployed ResNet-18 has 11.18M parameters and requires 1.814 GMACs.

**(a) Stage-specific training overhead**							
**Training Stage**	**Epochs**	**Student Views**	**Teacher Views**	**Epoch Time (min)**	**Stage Time (h)**	**Alloc. (GiB)**	**Reserved (GiB)**
Strong Clean	80	1	0	2.07	2.83	3.43	3.72
Mixed FT	30	2	0	3.65	1.96	4.66	5.91
Standard clean-view KD FT	24	1	1	2.85	1.20	3.72	5.14
CA-HOFT	24	2	1	4.63	1.98	5.87	7.02
Recovery FT	12	2	1	4.83	1.01	5.56	7.39
**(b) Deployment inference performance**							
**Method**		**Device**	**Precision**	**Threads**	**Latency (ms)**	**P95 (ms)**	**FPS**
CA-HOFT		RTX 4060 Laptop GPU	FP32	–	3.177 ± 0.070	5.119	314.73
CA-HOFT		Ryzen 7 7745HX CPU	FP32	8	14.308 ± 0.858	15.649	69.89

GPU denotes graphics processing unit; CPU denotes central processing unit; FP32 denotes 32-bit floating-point precision; FPS denotes frames per second; P95 denotes the 95th percentile; and GiB denotes gibibytes. The local environment comprised Windows 10, an NVIDIA GeForce RTX 4060 Laptop GPU (8 GB), an AMD Ryzen 7 7745HX CPU, Python 3.8.20, PyTorch 2.4.1+cu118, and CUDA 11.8. Training used automatic mixed precision, a batch size of 128, 12 workers, and identical storage conditions across stages. These controlled local profiles compare stage-specific training overhead; Alloc. and Reserved denote peak CUDA-allocated and CUDA-reserved memory, respectively. Deployment latency is reported as the mean ± sample standard deviation across repeat means. Image decoding, face detection, preprocessing, and host-to-device transfer are excluded.

**Table 12:** Source-reported model size, computation, inference components, and Occlusion-RAF-DB accuracy. Because definitions, units, profiling procedures, and evaluation sets differ across sources, the entries are descriptive and are not ranked. GFLOPs denotes giga floating-point operations. CA-HOFT is reported in GMACs, with one multiply–add counted as one MAC.

Method	Params (M)	Reported Computation	Extra Inference Components	Occlusion-RAF-DB Acc.
RAN [1]	300 ^†^	390 GFLOPs ^†^	Five regional forward passes and attention	82.72
Latent-OFER [3]	373 ^‡^	156 GFLOPs ^‡^	ViT-SVDD, reconstruction, and feature fusion	84.20
ORSANet [4]	60.2 ^§^	6.9 GFLOPs ^§^	Segmentation, landmarks, and semantic guidance	87.75
CA-HOFT (Ours)	11.18	1.814 GMACs	None	87.47

ViT-SVDD denotes Vision Transformer with Support Vector Data Description. ^†^ The RAN complexity is reproduced from the unified estimate in Table 7 of Latent-OFER [3]. ^‡^ Latent-OFER reports its own complexity. ^§^ ORSANet reports only the trainable portion of its model. These source-reported values are not normalized to a common counting convention and should not be interpreted as controlled efficiency ratios.

## Data Availability

The datasets analyzed in this study are RAF-DB, Occlusion-RAF-DB, AffectNet, Occl.-AffectNet, and FED-RO. They are available from their respective providers subject to the original access, use, and redistribution conditions; the corresponding source publications are cited in the manuscript. No new naturally captured dataset was collected in this study. The object-overlay RAF-DB benchmark is a derived synthetic evaluation set generated from the existing RAF-DB test split using author-owned RGBA occluder assets, as described in Section 4.1.

## Abbreviations

The following abbreviations are used in this manuscript:

FER	Facial Expression Recognition
CA-HOFT	Clean-Anchored Hard Occlusion Fine-Tuning
RAF-DB	Real-World Affective Faces Database
RAN	Region Attention Network
OADN	Occlusion-Adaptive Deep Network
FED-RO	Facial Expression Dataset with Real-World Occlusion
FT	Fine-Tuning
KD	Knowledge Distillation
CE	Cross-Entropy
KL	Kullback–Leibler Divergence
GPU	Graphics Processing Unit
CPU	Central Processing Unit
GMAC	Giga Multiply–Accumulate Operation
ViT-SVDD	Vision Transformer with Support Vector Data Description
